# Enabling efficient traceable and revocable time-based data sharing in smart city

**DOI:** 10.1186/s13638-021-02072-5

**Published:** 2022-01-04

**Authors:** Jiawei Zhang, Teng Li, Qi Jiang, Jianfeng Ma

**Affiliations:** 1 School of Cyber Engineering, Xidian University, Xi’an, China; 2 Network Communication Research Centre, Peng Cheng Laboratory, Shenzhen, China

**Keywords:** Attribute-based encryption, Revocability, Traceability, Time-based access control, Verifiable outsourced decryption

## Abstract

With the assistance of emerging techniques, such as cloud computing, fog computing and Internet of Things (IoT), smart city is developing rapidly into a novel and well-accepted service pattern these days. The trend also facilitates numerous relevant applications, e.g., smart health care, smart office, smart campus, etc., and drives the urgent demand for data sharing. However, this brings many concerns on data security as there is more private and sensitive information contained in the data of smart city applications. It may incur disastrous consequences if the shared data are illegally accessed, which necessitates an efficient data access control scheme for data sharing in smart city applications with resource-poor user terminals. To this end, we proposes an efficient traceable and revocable time-based CP-ABE (TR-TABE) scheme which can achieve time-based and fine-grained data access control over large attribute universe for data sharing in large-scale smart city applications. To trace and punish the malicious users that intentionally leak their keys to pursue illicit profits, we design an efficient user tracing and revocation mechanism with forward and backward security. For efficiency improvement, we integrate outsourced decryption and verify the correctness of its result. The proposed scheme is proved secure with formal security proof and is demonstrated to be practical for data sharing in smart city applications with extensive performance evaluation.

## Introduction

**Fig. 1:**
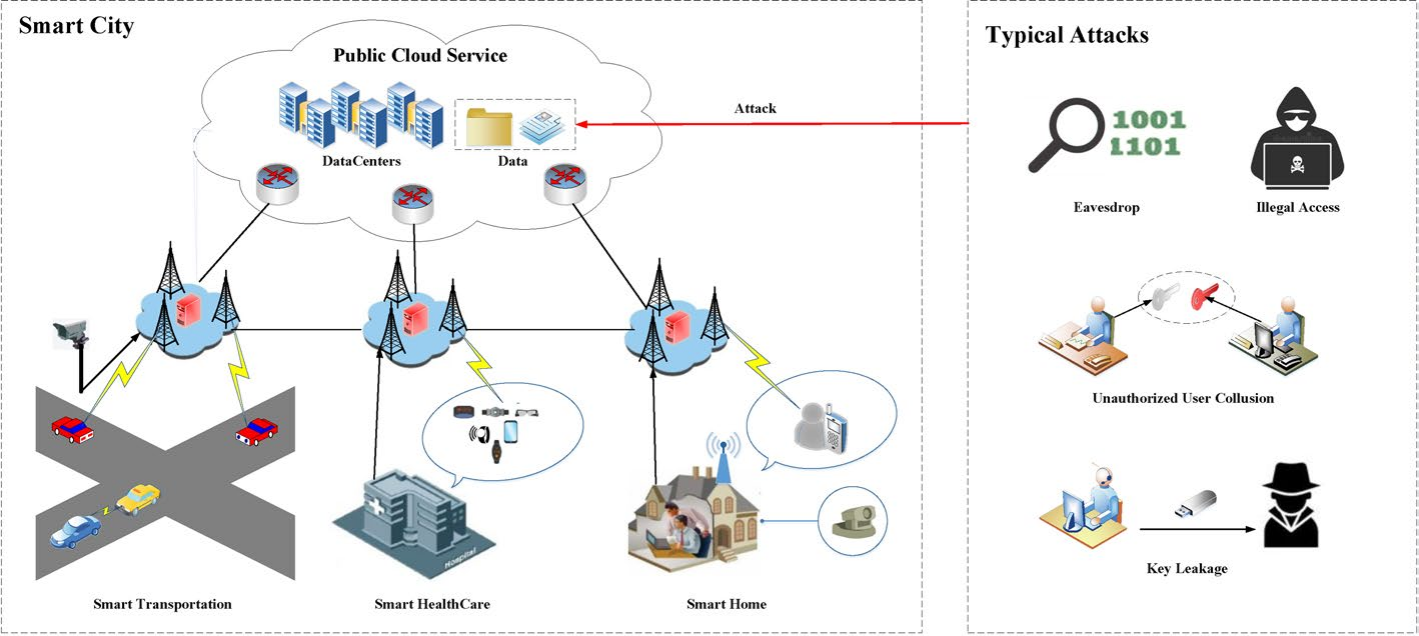
Data sharing scenario of smart city

As a well-accepted new service pattern, smart city is developing rapidly nowadays and facilitates the exponential growth of many novel applications, e.g., smart healthcare, smart campus, smart home, etc., which have attracted much attention and drives urgent demand for data sharing [1]. With the techniques of cloud computing and Internet of Things (IoT), these applications can easily gather massive valuable data from IoT devices and outsource the data to cloud for resource saving and data sharing [2]. Thus, users are able to access the shared application data with their intelligent terminals through mobile Internet all over the world [3]. In particular, this trend is being accelerated by the emerging fog computing (FC) [4] and 5G communication network [5] to realize low time delay, high-speed data transmission and massive access in service provision. Figure 1 shows a typical cloud-fog-based smart city data sharing scenario where application data are gathered from resource-poor IoT devices deployed all over the smart city and outsourced to the public cloud for storage, processing and sharing.

However, the shared data of smart city applications involve massive sensitive information and may inflict severe consequences on users if eavesdropped or illegally accessed. Let us take smart health care for example. The Personal Health Records (PHRs) essential for healthcare systems are collected from the wearable devices of patients and shared among healthcare practitioners for the diagnosis and treatment. The PHRs contain much private and sensitive information of patients (e.g., disease, identity, home address, etc.) [6] and will cause privacy leakage if eavesdropped and illegally accessed by attackers. Therefore, providing an efficient data access control scheme for data sharing in smart city applications permits no delay. A direct method to guarantee the security of the application data is encryption. However, it incurs heavy workload in key management and data distribution when multiple users access the shared data, especially in the setting of large-scale smart city applications. Fortunately, the promising ciphertext-policy attribute-based encryption (CP-ABE) [7] provides users with a fine-grained access control for their shared data by which users can designate desired access policy for their encrypted data.

As far as we know, many existing CP-ABE schemes [8–10] are vulnerable to malicious insiders who intend to leak their decryption key to outsiders for illicit profit pursuing, which poses a big issue on data security. It is intractable to trace these users according to the leaked decryption keys without specific identities. Meanwhile, it is of great necessity to afford a user revocation mechanism to enforce punishment on malicious users. Although the works in [11–14] propose several revocable CP-ABE schemes, they suffer from either low efficiency or the lack of forward and backword security (i.e., the revoked users cannot access the data shared before and after their revocation). Thus, how to efficiently trace a malicious user and enforce user revocation is one challenging issue. Furthermore, in many smart city applications, the shared data are time-sensitive in multi-user access; that is, some information should be accessed by an authorized user only after a specific time point, which is the second challenging issue to deal with. The scheme in [12] can address this requirement by introducing time-releasing encryption (TRE) into CP-ABE, but it suffers from high computational cost and is unsuitable for resource-poor terminals in applications of smart city, which brings the third challenging issue.

Therefore, in this paper, we concentrate on studying these challenging issues of data sharing in smart city applications and then present an efficient traceable and revocable time-based CP-ABE (TR-TABE) scheme to achieve efficient user tracing and revocation with forward and backward security together with time-based access control in data sharing. Combining with verifiable outsourced decryption, our scheme can achieve high computation efficiency and correctness verification considering semi-trusted fog nodes. Specifically, our main contributions are threefold:

- We propose an efficient traceable and revocable time-based data access control scheme for data sharing based on CP-ABE. All non-revoked users can access the shared data of their interests when and only when their attribute sets satisfy the access policy and their access time satisfy the designated time release policy at the same time.
- We design a new approach to achieve user tracing and revocation simultaneously. Based on white-box user tracing mechanism, the approach achieves efficient user tracing without maintaining a user list and the malicious user revocation with forward and backward security; that is, the revoked users cannot access the shared data before and after their revocation.
- To offset the heavy burden of computation in resource-poor devices, we introduce verifiable outsourced decryption into our scheme to offload a part of work task to semi-trusted fog nodes. Moreover, our scheme is resistant to key leakage attacks that an adversary cannot recover the ciphertext even he can compromise the transformation key.
- We present the formal security analysis for the proposed scheme to show that it achieves the security goals. Besides, we implement our scheme and conduct extensive experimental simulation to demonstrate its efficiency and practicality in data sharing of smart city applications.

The rest of this paper is outlined as follows. Some related work is reviewed in Sect. 2. In Sect. 3, we give several notations and definitions used in this paper. The system model, formal definition and security model and concrete constructions of our scheme are presented in Sect. 4. In Sect. 5, we present the construction of our proposal and discuss detailed security analysis with performance evaluation of our scheme. Finally, we make a conclusion for our work in Sect. 6.

## Related work

*Ciphertext-policy ABE*. As considered to be a promising technique for data access control, attribute-based encryption (ABE) was first proposed in [15]. Later, Goyal et al. [7] further studied ABE and divided it into two types: ciphertext-policy ABE (CP-ABE) and key-policy ABE (KP-ABE). In the former, user can flexibly designates the access policy for ciphertext. Subsequently, a great many studies were dedicated to CP-ABE [16–20]. On account of the low efficiency in decryption, many researchers proposed outsourced CP-ABE, such as [21–27]. These schemes offload most of the complex computation in decryption to third party, such as cloud and fog node, to save cost for resource-poor devices. Very recently, some researches have introduced time-sensitive data access control for time-based applications by combining timed release encryption (TRE) [28] with data access control schemes, such as [29], but they only achieved coarse granularity. Later, Hong et al. [12] proposed a time-sensitive CP-ABE scheme with fine-grained access control, but it still incurs heavy cost in decryption.

*Revocable ABE*. Revocable ABE has two directions: One is attribute revocation [30] that dynamically revokes one or more user attributes and the other is user revocation that revokes all of a user’s permission directly. In user revocation, there are also two classifications: One is direct revocation and the other is indirect revocation. The studies in [11, 31] proposed several direct user revocable CP-ABE schemes, but they bring about complex computation. Later, Liu et al. [32] made some improvement by setting valid time period for user secret key. Recently, Xiong et al. [33] have proposed a user revocable CP-ABE scheme with broadcast encryption to achieve direct user revocation, while it incurs high communication cost and cannot achieve ciphertext update. Very recently, the work in [34] introduces ciphertext update into direct user revocation. In the meantime, Lee et al. [35] raised a revocable CP-ABE scheme with self-update to achieve indirect user revocation, but it introduces much computation cost. Later, Cui et al. [36] proposed a server-aided indirect user revocable CP-ABE scheme, but it is vulnerable to decryption key leakage attack. To address the problem, the proposals in [37, 38] made corresponding contributions based on [39], but their computation cost is still high.

*Traceable ABE*. To achieve user traceability in CP-ABE schemes, Li et al. [40] proposed the first accountable CP-ABE scheme to resist user collusion for key sharing. Then, the scheme [41] extends [40] to multi-authority setting. However, both the schemes only support AND gate policy. Later, the scheme in [42] proposed an accountable multi-authority scheme that can support access policy tree, but this kind of schemes cannot support effective user tracing. On this account, the schemes in [43, 44] presented black-box traceable CP-ABE schemes, but they incur a large size of public parameters and ciphertexts. To address the bottleneck issue of black-box traceability, Ning et al. [45] proposed a large universe traceable CP-ABE scheme without any identity table by introducing white-box tracing mechanism into CP-ABE. Based on this scheme, Liu et al. [46] proposed an efficient traceable CP-ABE scheme with user revocation which lacks of forward and backward security, while Yan et al. [47] proposed another traceable CP-ABE scheme supporting multi-domain setting and full security in standard model, but it is implemented over composite order groups and suffers from heavy computation burden.

## Preliminary

The section presents several relevant notions and definitions employed in our paper.

### Notations

In this paper, $\{1,\ldots , n\}$ is abbreviated to [*n*], an integer set modulo a prime number *p* is denoted by $Z_p$, user revocation list by ${\mathrm{RL}}_u$, user secret key, update key and transformation key by ${\mathrm{SK}}_{\mathrm{ID}}, {\mathrm{UK}}_{t}, {\mathrm{DK}}_{{\mathrm{ID}},t}$, respectively.

### Access structure

#### **Definition 1**

(*Access structures* [26]). Suppose $\{L_1,\ldots ,L_n\}$ is a parties set. One of the collection $L \subseteq 2^{\{L_1,\ldots ,L_n\}}$ is considered to be monotone $\text {if} \forall M,N: M \in L \text {and} M \subseteq M, \text {then} N \in L$. An access structure that is monotone is defined as one of the non-empty subsets *L* of $\{L_1,\ldots ,L_n\}, i.e., L \subseteq 2^{\{L_1,\ldots ,L_n\}}\backslash {\varnothing }$. The elements in *L* are defined as authorized sets and the other sets are defined as unauthorized sets. Without loss of generality, we can describe users with their attribute set.

### Access policy tree

#### **Definition 2**

(*Access Tree* [27]). Similar to [27], suppose *R* is a policy tree with each node $x \in R$, where we use a threshold gate to represent non-leaf nodes and a leaf node is an attribute ${\mathrm{att}}(x)$. As to a threshold gate $x \in R$, we use ${\mathrm{num}}(x)$ which is the number of children and the threshold value ${\mathrm{th}}_x \in [1, {\mathrm{num}}(x)]$ to depict it. Specifically, if ${\mathrm{th}}_x = 1$, it is an OR gate, and if ${\mathrm{th}}_x = {\mathrm{num}}(x)$, it is an AND gate. If $x \in T$ is a leaf node, its threshold value is ${\mathrm{th}}_x = 1$.

Moreover, suppose $r \in R$ is the root node. If $x \in T$ is a non-leaf node, ${\mathrm{child}}(x)$ is a collection of its children and ${\mathrm{parent}}(x)$ denotes the parent node of *x*. Thus, we can infer that $|{\mathrm{child}}(x)| = {\mathrm{num}}(x)$. We use the function ${\mathrm{index}}(x)$ to signify the unique index value of each node $x \in T$.

*Access tree satisfaction*. Suppose *R* is an access tree rooted from node *r*, then we use $R_x$ to denote a subtree rooted from node $x \in R$. Here, we define $R_x({\mathcal {A}}) = 1$ when and only when ${\mathcal {A}}$ (a attribute set) is satisfactory to the subtree $R_x$; that is, when a leaf node *x* has ${\mathrm{att}}(x) = {\mathrm{att}}_i \in {\mathcal {A}}$, then $R_x({\mathcal {A}}) = 1,$ and when a non-leaf node has $\forall z_x \in {\mathrm{child}}(x)$, the number of *z* satisfying $R_z({\mathcal {A}}) = 1$ exceeds ${\mathrm{th}}_x$, $R_x({\mathcal {A}}) = 1$.

### KUNode algorithm

As in [6], we utilize a binary tree for user revocation. Let BT be a complete binary tree. It has $N_u$ leaf nodes and a root node RN. For each non-leaf node $\delta$, its left and right child node is denoted as $\delta _{\mathrm{l}}$ and $\delta _{\mathrm{r}}$. Each leaf node $\theta$ is associated with a user ID. Path(ID) denotes the nodes in the path from RN to $\theta$. ${\mathrm{RL}}_u$ is the revocation list storing the tuple $({\mathrm{ID}}, t)$ with revoked user ID at time period *t*. The algorithm ${\mathrm{KUNode}}({\mathrm{BT}}, {\mathrm{RL}}_u, t)$ can obtain a minimum node set $Y_b$. For any element $({\mathrm{ID}}, t) \in {\mathrm{RL}}_u, {\mathrm{Path(ID)}} \bigcap Y_b = \emptyset$. For other element $({\mathrm{ID}},t) \notin {\mathrm{RL}}_u$, the algorithm can get a node $\delta \in Y_b$ that is an ancestor of the leaf node $\theta$ related to the user ID. The detail of this algorithm is shown in 1. 
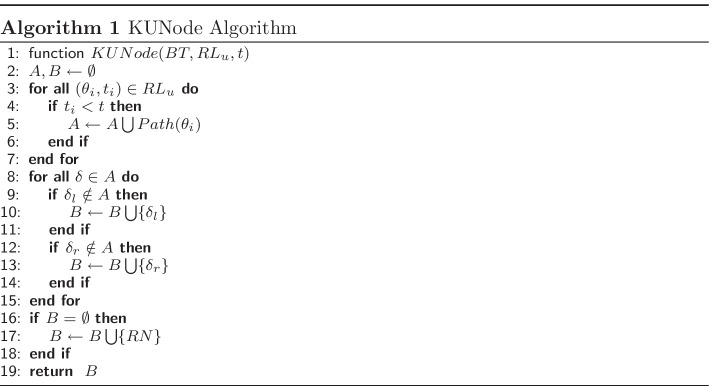


### Time binary tree

#### **Definition 3**

(*Time Binary Tree* [39]). To guarantee forward security, the system lifetime has $N_t = 2^d$ discrete time period denoted by $\{0,1,\ldots , N_t - 1\}$. The lifetime is represented by a binary time tree ${\mathcal {T}}_t$ of depth *d*. For each leaf node $\sigma _t$ in time tree ${\mathcal {T}}_t$, it is assigned with a time period *t* in chronological left-to-right order. For each node $\sigma \in {\mathcal {T}}_t$, ${\mathrm{RC}}_{\sigma }$ denotes its right child, and $b_{\sigma }$ indicates the binary string according to the path from the root node to $\sigma$, where the path traverses the left child and right child of the parent node is denoted by 0 and 1, respectively. Given a time period *t*, ${\mathcal {N}}_t = \{{\mathrm{RC}}_{\sigma }|\sigma \in {\mathrm{Path}}(\sigma _t) \wedge {\mathrm{RC}}_{\sigma } \notin {\mathrm{Path}}(\sigma _t)\} \cup \{\sigma _t\}$ which has the property that $\forall {\hat{t}} > t$, for each $\sigma \in {\mathcal {N}}_t$, a node $\sigma ^{'} \in {\mathcal {N}}_{{\hat{t}}}$ exists that $b_{\sigma }$ is a prefix of $b_{\sigma ^{'}}$.

### Cryptographic background

Here, we give the definition of some cryptographic primitives including bilinear map, decisional bilinear Diffie–Hellman exponent (BDHE) assumption and *l*-strong Diffie–Hellman assumption.

#### **Definition 4**

(*Bilinear Maps* [27]): We consider two *p*-ordered $G_0$ and $G_1$ groups that are multiplicative cyclic, where *p* is a prime. $\varepsilon , \epsilon$ are two generators of group $G_0$. If the may ${\hat{e}} :G_0 \times G_0 \rightarrow G_1$ satisfies the following properties:

1. Bilinearity: $${\hat{e}} (\varepsilon ^a,\epsilon ^b)= {\hat{e}} (\varepsilon ,\epsilon )^{ab}, \forall a,b \in Z_p, \varepsilon ,\epsilon \in G$$.
2. Non-Degeneracy: ${\hat{e}}(\varepsilon ,\epsilon )\ne 1_{G_1}$, ${\hat{e}}(\varepsilon , \varepsilon )$ is a generator of $G_1$.
3. Computability: ${\hat{e}}(\varepsilon , \epsilon )$ is efficiently computable for all $\varepsilon ,\epsilon \in G_0$,

then we call it a bilinear map.

#### **Definition 5**

(*Decisional Bilinear Diffie–Hellman (DBDH) Assumption* [30]): Given two cyclic groups *E* and *F* and their orders are both the prime *p*. Suppose a generator $h \in E$ and a bilinear mapping ${\hat{e}}: E \times E \rightarrow F$. The DBDH problem is defined to find out the difference between ${\hat{e}}(h,h)^{c d m}$ and ${\hat{e}}(h,h)^{\nu }$ on inputting the tuple $(h, h^{c}, h^{d}, h^{m})$, where $c, d, m, \nu \in _R Z_p$.

It is considered that DBDH assumption holds when no probabilistic polynomial time (PPT) adversaries can deal with the DBDH problem whose advantages are non-negligible.

#### **Definition 6**

(*l*-*SDH assumption* [46]). Assuming a $(l+1)$-tuple $(h, h^x, h^{x^2}, \ldots , h^{x^l})$, the *l*-SDH problem is to output a pair $(c, h^{1/1+c}) \in Z_p \times G_x$. If $|P_r[{\mathcal {A}}(h, h^x, h^{x^2}, \ldots , h^{x^l}) = (c, h^{1/x+c})]| \ge \varepsilon$ for an algorithm ${\mathcal {A}}$ randomly choosing $x \in Z_p$; then, the advantage for an algorithm solving the problem is $\varepsilon$.

If no PPT algorithm ${\mathcal {A}}$ can solve the *l*-SDH problem with the advantage at least $\varepsilon$, then the *l*-SDH assumption holds.

## Methods

We present the concrete construction of our TR-TABE scheme together with the system model, formal definition and security model for our proposal in this section.

### System model and threat model

**Fig. 2:**
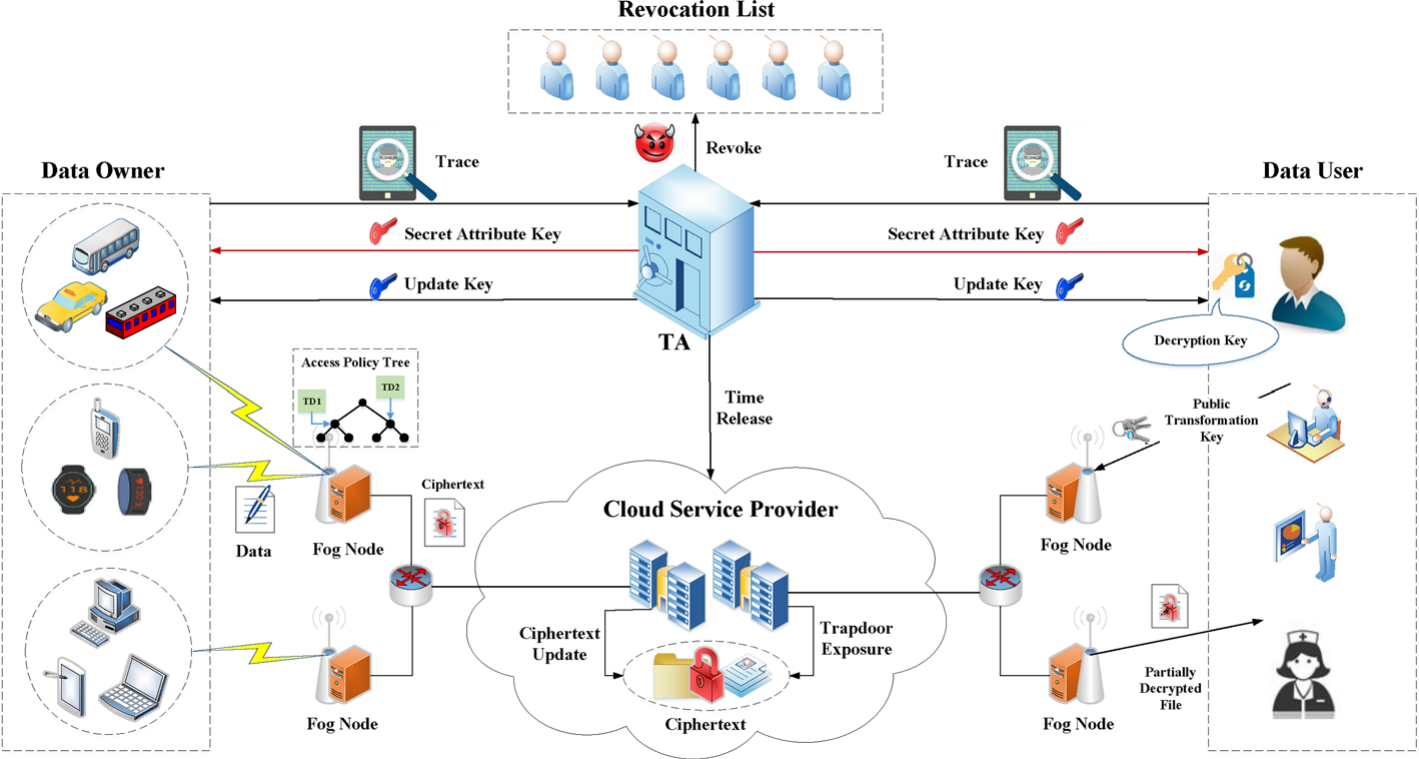
Framework of TR-TABE scheme

As shown in Fig. 2, the system involves five entities, i.e. Trusted Authority (TA), Cloud Service Provider (CSP), Fog Node (FN), Data Owner (DO) and Data User (DU), which are described as follows:

- **TA**: The entity is in charge of initiating the entire system by generating system public key and master key. It also responsible for user tracing and revocation, in which TA exposes the real identity of a malicious user who leaked his secret key and revokes the user by adding his identity into user revocation list.
- **CSP**: The entity is responsible for providing enormous storage and computation resources for customers. It also offers a large number of services for users, such as data sharing and data outsourcing services. Moreover, it is in charge of the trapdoors exposure and update for the shared ciphertexts in it.
- **FN**: The entity is in charge of user requests processing and conducting outsourced decryption for users who accessing the shared data in CSP.
- **DO**: The entity takes charge of generating ciphertexts according to access policy and current time period for his own data to be stored and shared in CSP.
- **DU**: The entity can access desired data shared in CSP and receive partially decrypted ciphertexts from FN. He can recover the correct plaintext if he is authorized and not revoked at current period. The entity is also responsible for generating his own decryption key by combining update key and his secret attribute key.

In our proposal, TA and DO are considered to be fully trusted. CSP and FN are supposed to be semi-trusted who conduct their protocol honestly but are curious about the contents transmitted. The entity DU involves untrusted users that may initiate collusion attack and conduct malicious activities for extra interest revenues, such as share his decryption key to outsiders.

On account of these threats, we take the following design goals into consideration for our scheme:

- *User Collusion Resistance*. The scheme should prevent unauthorized users colluding with each other to obtain more privileges for accessing the shared data.
- *Time-based and Fine-Grained Access*. The data shared in CSP should not be learned by any malicious third party and can be accessed only by authorized DU after specific time point according to designated access policy.
- *Secure User Revocation*. Any malicious cloud users that conduct malicious activities should be revoked with forward and backward security; that is, they cannot access the data shared before and after their revocation.
- *Traceability*: Any malicious cloud users that illegally leaked their decryption keys for profit should be precisely recognized and exposed their real identity efficiently.
- Verifiability: Due to the semi-trusted fog nodes, DU should have the ability to verify the correctness of the results from outsourced decryption.
- Efficiency: As the resource-poor mobile devices deployed and utilized in smart city, it is preferable for DU to outsource the highly computational burden in decryption to CSP for efficiency improvement.

### The formal definition of TR-TABE

Our TR-TABE scheme involves the following algorithms:

- ${\mathrm{Setup}}(\lambda , N_u, d) \rightarrow \{{\mathrm{PK}}, {\mathrm{MSK}}\}$: TA is responsible for the algorithm execution. Given the security parameters $\lambda , N_u, d$, the algorithm constructs the whole system for the generation of system public key *PK* that is published publicly and master key MSK stored secretly.
- ${\mathrm{AKeyGen}}({\mathrm{PK, sta, ID, S}}) \rightarrow {\mathrm{SK}}_{{\mathrm{ID}}}$: TA is in charge of the algorithm execution. Given the system public key PK, state information sta, user identity ID and his attribute set *S*, the algorithm outputs the secret attribute key and sends it to user ID through secure channel.
- ${\mathrm{UKeyGen}}({\mathrm{PK}}, {\mathrm{MSK}}, {\mathrm{RL}}_u, {\mathrm{sta}}, t) \rightarrow {\mathrm{UK}}_{t}$: TA is responsible for the execution of this algorithm. Given the system public key PK and master key MSK, user revocation list ${\mathrm{RL}}_u$, state information sta and current time period *t*, the algorithm returns the update key ${\mathrm{UK}}_{t}$ of current time period *t*.
- ${\mathrm{DKeyGen}}({\mathrm{PK}}, {\mathrm{SK}}_{{\mathrm{ID}}}, {\mathrm{UK}}_{t}) \rightarrow {\mathrm{DK}}_{{\mathrm{ID}},t}$: Non-revoked user is responsible to execute the algorithm. Given the system public key PK, original secret attribute key ${\mathrm{SK}}_{{\mathrm{ID}}}$ and update key ${\mathrm{UK}}_t$, the algorithm combines ${\mathrm{SK}}_{{\mathrm{ID}}}$ with ${\mathrm{UK}}_t$ to derive his decryption key ${\mathrm{DK}}_{{\mathrm{ID}},t}$ at the beginning of each time period.
- ${\mathrm{Encrypt}}({\mathrm{PK}}, m, t, {\mathcal {T}}_a) \rightarrow {\mathrm{CT}}_t$: The algorithm is in the charge of DO. Given the system public key PK, the message *m*, current time period *t* and access policy tree ${\mathcal {T}}_a$, the algorithm outputs the ciphertext of *m* according to ${\mathcal {T}}_a$ at time period *t* and uploads it to CSP.
- ${\mathrm{CTUpdate}}({\mathrm{PK}}, {\mathrm{CT}}_t, {\hat{t}}) \rightarrow {\mathrm{CT}}_{{\hat{t}}}$: CSP is responsible for the execution of this algorithm. Given the system public key PK, ciphertext ${\mathrm{CT}}_t$ of time period *t* and the next time period ${\hat{t}} > t$, the algorithm updates the ciphertext components associated with the symmetric encryption key from current time period *t* to the next time period ${\hat{t}}$ and outputs ${\mathrm{CT}}_{{\hat{t}}}$.
- ${\mathrm{TokenGen}}({\mathrm{PK}}, t) \rightarrow {\mathrm{TK}}_t$: The algorithm is in the charge of TA. Given the system public key PK and time period *t*, the algorithm outputs the time token ${\mathrm{TK}}_t$.
- ${\mathrm{Trap}}({\mathrm{PK}}, {\mathrm{TK}}_t) \rightarrow {\mathrm{TP}}_{\sigma ,x}^{'}$: CSP is responsible for the execution of this algorithm. Given the system public key PK and the time token ${\mathrm{TK}}_t$ of time period *t*, the algorithm outputs the exposed trapdoor ${\mathrm{TP}}_{\sigma ,x}^{'}$ of related ciphertexts and replaces the corresponding ciphertext components with it.
- ${\mathrm{TKeyGen}}({\mathrm{PK}}, {\mathrm{DK}}_{{\mathrm{ID}},t}) \rightarrow {\mathrm{TK}}_{{\mathrm{ID}},t}$: The algorithm is in the charge of DU. Given the system public key PK and decryption key ${\mathrm{DK}}_{{\mathrm{ID}},t}$ of time period *t*, the algorithm outputs the transformation key ${\mathrm{TK}}_{{\mathrm{ID}},t}$ for outsourced decryption.
- ${{\mathrm{Decrypt}}}_{{\mathrm{OUT}}}({\mathrm{PK}}, {\mathrm{TPK}}_{{\mathrm{ID}},t}, {\mathrm{CT}}_t, t) \rightarrow {{\mathrm{CT}}_t}^{'}$: CSP is responsible for the algorithm execution. Given the system public key PK, public transformation key ${\mathrm{TPK}}_{{\mathrm{ID}},t}$, ciphertext ${\mathrm{CT}}_t$ and time period *t*, the algorithm finishes outsourced decryption and outputs partially decrypted ciphertext ${{\mathrm{CT}}_t}^{'}$.
- ${{\mathrm{Decrypt}}}_U ({\mathrm{PK}}, {\mathrm{TSK}}_{{\mathrm{ID}},t}, {\mathrm{CT}}_{\sigma _t}^{'}) \rightarrow \text {m or null}$: DU is in charge of the execution of this algorithm. Given the system public key PK, secret transformation key ${{\mathrm{TSK}}_{\mathrm{ID}},t}$ and partially decrypted ciphertext ${{\mathrm{CT}}_t}^{'}$, the algorithm recovers and outputs the plaintext message *m* if authorized and null if not authorized.
- ${\mathrm{UTrace}}({\mathrm{PK}}, {\mathrm{DK}}_{{\mathrm{ID}},t}, {\mathrm{RL}}_u, t) \rightarrow {\mathrm{ID}} \text { or } {\mathrm{null}}$: The algorithm is in the charge of TA. Given the system public key PK, decryption key ${\mathrm{DK}}_{{\mathrm{ID}},t}$, revocation list ${\mathrm{RL}}_u$ and time period *t*, the algorithm exposes the identity ID of malicious user and adds it into revocation list ${\mathrm{RL}}_u$.

### Security model

#### Traceability of TR-TABE

The traceability of our scheme is modeled through a security game between adversary ${\mathcal {A}}$ and challenger ${\mathcal {C}}$ as follows:

*Init*: ${\mathcal {C}}$ executes Setup algorithm to initiates the system public key PK to ${\mathcal {A}}$.

*Key Query*: ${\mathcal {A}}$ conducts *q* queries for decryption key generation given $({\mathrm{ID}}_1, S_1), \ldots , ({\mathrm{ID}}_q, S_q)$ that satisfies ${\mathrm{ID}}_i \in {\mathrm{RL}}_u$ or ${\mathcal {T}}_a(S_i) \ne 1$ for $i \in [q]$. Then, ${\mathcal {C}}$ runs DKeyGen to derive decryption keys for ${\mathcal {A}}$. For a better understanding, we assume all decryption are generated at time period *t*.

*Key Forgery*: ${\mathcal {A}}$ outputs a decryption key ${\mathrm{DK}}_{{\mathcal {A}}, t}$.

If ${\mathcal {A}}$ can win the game, then for a well-formed decryption key ${\mathrm{DK}}_{{\mathcal {A}}, t}$, ${\mathrm{UTrace}}({\mathrm{PK}}, {\mathrm{RL}}_u, {\mathrm{DK}}_{{\mathcal {A}}, t}, t) \ne {\mathrm{null}}$ and ${\mathrm{UTrace}}({\mathrm{PK}}, {\mathrm{RL}}_u, {\mathrm{DK}}_{{\mathcal {A}}, t}, t) \notin \{{\mathrm{ID}}_1, \ldots , {\mathrm{ID}}_q\}$. Thus, the advantage of ${\mathcal {A}}$ in winning the game is:

$$\begin{aligned} \begin{aligned} {\mathrm{Adv}}_{{\mathcal {A}}} = P_r[{\mathrm{UTrace}}({\mathrm{PK}}, {\mathrm{DK}}_{{\mathcal {A}}, t}, {\mathrm{RL}}_u, t) \notin {\hat{Y}} \bigcup \{{\mathrm{ID}}_1, \ldots , {\mathrm{ID}}_q\}] \end{aligned} \end{aligned}$$

where ${\hat{Y}}$ is the leaf nodes in $\mathcal {BT}$ covered by ${\mathrm{KUNode}}(\mathcal {BT}, {\mathrm{RL}}_u, t)$.

##### **Definition 7**

The TR-TABE has traceability if no PPT adversary can break the above game with a non-negligible advantage.

#### IND-CPA security of TR-TABE

According to the ability of adversary, there are two types of adversaries: Type-*A* adversary who has insufficient privilege for data access, even he is not revoked or arrives at release time and Type-*B* adversary who has enough rights but has been revoked before declared time period. Then, we describe the IND-CPA security model for our TR-TABE scheme corresponding to these adversaries and conduct a selective security game between an adversary ${\mathscr {A}}$ and a challenger ${\mathscr {C}}$. specified as follows:

*Init*: ${\mathscr {A}}$ sends a challenge access policy tree ${\mathcal {T}}_a^*$, a time period $t^*$ and a revocation list ${\mathrm{RL}}_u^*$ to ${\mathscr {C}}$.

*Setup*: ${\mathscr {C}}$ executes the Setup algorithm of our scheme and outputs the public parameters to ${\mathscr {A}}$.

*Phase 1*: ${\mathscr {A}}$ issues a polynomial number of queries $\{q_i\}_{i \in [q]}$, where $q_i$ belongs the following:

- *SK Query*: ${\mathscr {A}}$ requests ${\mathscr {C}}$ for secret key with an attribute set *S*. As a response, ${\mathscr {C}}$ outputs the secret key and returns them to ${\mathscr {A}}$.
- *UK Query*: ${\mathscr {A}}$ requests ${\mathscr {C}}$ for update key with a revocation list ${\mathrm{RL}}_u$ and time period *t*. As a response, ${\mathscr {C}}$ outputs the update key and returns it to ${\mathscr {A}}$. Besides, ${\mathscr {C}}$ issue a decryption key query to get decryption key for algorithm execution.
- *DK Query*: ${\mathscr {C}}$ running decryption key generation algorithm after *SK Query* and *UK Query* to get decryption key and sends it to ${\mathscr {A}}$.
- *TK Query*: ${\mathscr {A}}$ issues queries for transformation key similar to that in *SK Query*. ${\mathscr {C}}$ executes TKeyGen to generates transformation key pairs and sends it to ${\mathscr {A}}$.
- *RL Query*: ${\mathscr {A}}$ submits user revocation request with identity ID at time period *t*, ${\mathscr {C}}$ updates the revocation list.

Note that all the above queries have the same constraints as that of [6]; that is, *RK Query* and *UK Query* must be queried later than previous queries and they cannot be queried at the same time period. *SK Query* cannot be queried before *UK Query* at the same time period.

*Challenge*: ${\mathscr {A}}$ finishes the above phase and issues two equal-length data $B_0$ and $B_1$ to ${\mathscr {C}}$. Then, ${\mathscr {C}}$ randomly picks a bit $\epsilon \in [0,1]$ and encrypts $m_{\epsilon }$ according to ${\mathcal {T}}_a^*$ and $t^*$ and sends it to ${\mathscr {A}}$.

*Phase 2*: It is similar to *Phase 1* with the same constraints.

*Guess*: ${\mathscr {A}}$ publishes his guess $\epsilon ^{'}$ for $\epsilon$. If $\epsilon ^{'} = \epsilon$, he wins the security game. The advantage of ${\mathscr {A}}$ is defined as ${\mathrm{Adv}}_{{\mathscr {A}}} = |{\mathrm{Pr}}[\epsilon ^{'} = \epsilon ] - \frac{1}{2}|$.

##### **Definition 8**

A TR-TABE scheme is indistinguishable against chosen-plaintext attack (CPA) if all probabilistic polynomial adversaries cannot break the security game.

### Overview of our proposal

**Fig. 3:**
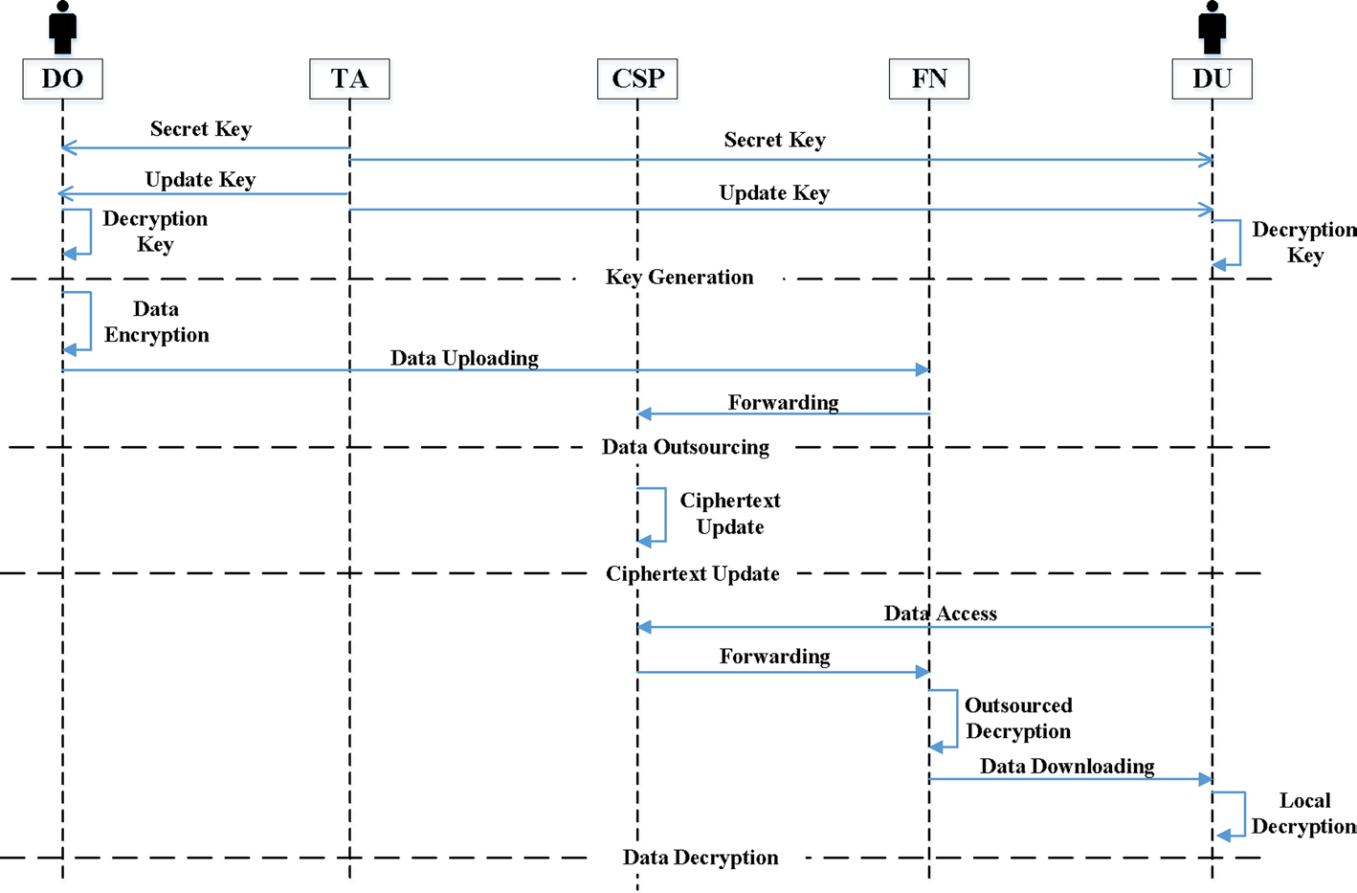
Procedure of TR-TABE scheme

In our proposal, we intend to achieve time-based and fine-grained access control for data sharing in applications of smart city with efficient user tracing and revocation over large attribute universe. Our scheme holds constant sized public parameters and can trace malicious users who try to share their decryption keys with outsiders for profit then revoke them. Any revoked users cannot access ciphertexts before and after his revocation and the ciphertext update requires no secret keys. Moreover, based on our system model, we give an overview for our TR-TABE scheme involving the following four phases shown in Fig. 3.

- Initialization. In this phase, TA initiates the whole system by generating system public parameters and master key for the system. All entities in the system can obtain the system public parameters.
- Key Generation. In this phase, TA creates secret attribute keys for users according to their attribute set and periodically distributes update keys at the beginning of each time period. Unrevoked users can calculate their decryption key with secret key and update key.
- Encryption. In this phase, DO encrypts the sensitive and important data using symmetric encryption algorithm before uploading them to the CSP. Moreover, DO designates an access policy for the data to enable fine-grained access control and data sharing in CSP. Moreover, CSP periodically updates outsourced ciphertexts at the end of each time period.
- Decryption. In this phase, DU requests desired data from CSP through FN. After outsourced decryption, FN returns the DU with the partially decrypted ciphertexts. DU recovers the plaintext according to the result of verification.
- Trace. In this phase, if any malicious cloud user leaks his decryption key for illegal profits, TA will obtain the identity of key owner according to the exposed decryption key and conduct revocation.

### Construction

#### Initialization phase

- ${\mathrm{Setup}}(\lambda , N_u, d) \rightarrow \{{\mathrm{PK}}, {\mathrm{MSK}}\}$: On inputting the security parameter $\lambda$, the maximum number of system users $N_u$ and the depth *d* of the time tree ${\mathcal {T}}_t$, the setup algorithm initiates a binary tree $\mathcal {BT}$ with $N_u$ leaf nodes, the user revocation list ${\mathrm{RL}}_u = \emptyset$ and system initial state ${\mathrm{sta}} = \mathcal {BT}$ for user revocation mechanism. It also computes the total number of time periods $N_t = 2^d$ in ${\mathcal {T}}_t$, where the set of total time periods is denoted by $F_T$. Then, it generates two multiplicative bilinear groups $G_0$ and $G_1$ of prime order *p* with a generator *g* of group $G_0$ and a bilinear map ${\hat{e}}: G_0 \times G_0 \rightarrow G_1$ and selects random numbers $\alpha , a, \gamma \in Z_p$. The algorithm also chooses $\eta , \eta _1, \eta _2, \cdots , \eta _d \in _R G_0$ and a probabilistic symmetric encryption scheme (Enc, Dec) from a binary string to $Z_p^*$ with a random key ${\hat{K}} \in {\mathcal {K}}$. Then, it selects collision-resistant hash functions $H_0 : G_1 \rightarrow {\mathcal {K}}, H_1:\{0,1\}^{*} \rightarrow G_0, H_2: G_1 \times {\mathcal {M}} \rightarrow {\mathcal {M}}$, where $H_0,H_1$ are two collision-resistant hash functions, $H_2$ is a pseudorandom function, ${\mathcal {K}}$ is the symmetric encryption key universe and ${\mathcal {M}}$ is the message universe. The algorithm also defines a function $H: F_T \rightarrow G_0$ as $H(t) = \eta \prod _{i=1}^{d} \eta _i^{t_i}$ for each time period $t \in {\mathcal {T}}_t$ expressed by a *d*-length binary string, where $t_i$ denotes the *i*th bit of *t*. Next, it computes and outputs the system public key and master key as follows:
  $$\begin{aligned} \begin{aligned} {\mathrm{PK}}&= (G_0, G_1, {\hat{e}}, g, g^a, {\hat{e}}(g,g)^{\alpha }, \eta _1, \ldots , \eta _d, h = g^{\gamma }, ({\mathrm{Enc}}, {\mathrm{Dec}}), H, H_0, H_1, H_2) \\ {\mathrm{MSK}}&= (g^{\alpha }, a, \gamma , {\hat{K}}) \end{aligned} \end{aligned}$$
  Finally, TA publishes PK publicly and stores MSK locally in secret.

#### Key generation phase

- ${\mathrm{AKeyGen}}({\mathrm{PK}}, {\mathrm{sta}}, {\mathrm{ID}}, S) \rightarrow {\mathrm{SK}}_{{\mathrm{ID}}}$: The algorithm chooses a random empty leaf node $\theta$ of the binary tree $\mathcal {BT}$ to store user ID. For each node $\delta \in {\mathrm{Path}}(\theta )$, the algorithm selects a random element $\mu _{\delta } \in G_0$ if the node $\delta$ is not assigned and a random number $r_{\delta } \in Z_p$. Then, the algorithm calculates $c = {\mathrm{Enc}}({\mathrm{ID}}, {\hat{K}})$. It also chooses a random number $r_{{\mathrm{ID}}} \in Z_p$ for user ID and $r_i \in Z_p$ for each attribute ${\mathrm{att}}_i \in S$ and computes
  $$\begin{aligned} \begin{aligned} {\mathrm{SK}}_{\delta , {\mathrm{ID}}}&= \{{\mathrm{sk}}_{\delta , 0} = g^{r_{\delta }}, {\mathrm{sk}}_{\delta , 1} = \mu _{\delta } \cdot g^{{\mathrm{ar}}_{\delta }}, {\mathrm{sk}}_{\delta , 2} = g^{\frac{\alpha + r_{{\mathrm{ID}}}}{a + c}} g^{r_{\delta }}, \\ \{{\mathrm{sk}}_{i,1}&= g^{r_{{\mathrm{ID}}}} H_1({\mathrm{att}}_i)^{r_i}, {\mathrm{sk}}_{i,2} = g^{r_i}, {\mathrm{sk}}_{i,3} = g^{{\mathrm{ar}}_i}\}_{{\mathrm{att}}_i \in S}\} \end{aligned} \end{aligned}$$
  Finally, the algorithm outputs the secret attribute key of user ID as ${\mathrm{SK}}_{{\mathrm{ID}}} = \{S, K_{{\mathrm{ID}}} = c, \{{\mathrm{SK}}_{\delta , {\mathrm{ID}}}\}_{\delta \in {\mathrm{Path}}(\theta )}\}$ with updated system state sta.
- ${\mathrm{UKeyGen}}({\mathrm{PK}}, {\mathrm{MSK}}, {\mathrm{RL}}_u, {\mathrm{sta}}, t) \rightarrow {\mathrm{UK}}_{t}$: Given the current time period *t*, the algorithm run ${\mathrm{KUNode}}(\mathcal {BT}, {\mathrm{RL}}_u, t)$ to get the node set *Y*. For each node $\delta \in Y$, the algorithm fetches $\mu _{\delta }$. If $\delta$ is not assigned previously, it assigns a random $\mu _{\delta } \in G_0$ to the node $\delta$. Then, the algorithm chooses a random number $\beta _{\delta } \in Z_p$ for each node $\delta$ and computes
  $$\begin{aligned} \begin{aligned} {\mathrm{UK}}_{\delta ,t} = \{{\mathrm{uk}}_{\delta , t,0} = \mu _{\delta }^{-1} \cdot H(t)^{\beta _{\delta }}, {\mathrm{uk}}_{\delta , t, 1} = g^{{\beta _{\delta }}}\} \end{aligned} \end{aligned}$$
  Finally, the algorithm outputs the update key ${\mathrm{UK}}_{t} = \{{\mathrm{UK}}_{\delta }\}_{\delta \in Y}$ of current time period *t*.
- ${\mathrm{DKeyGen}}({\mathrm{PK}}, {\mathrm{SK}}_{{\mathrm{ID}}}, {\mathrm{UK}}_{t}) \rightarrow {\mathrm{DK}}_{{\mathrm{ID}},t}$: On inputting the secret attribute key ${\mathrm{SK}}_{{\mathrm{ID}}}$ and update key ${\mathrm{UK}}_{t}$ for current time period *t*of user ID, the algorithm calculates the decryption key for non-revoked user *ID* in the current period *t*.
  - It first obtains the unique node ${\hat{\delta }} = {\mathrm{Path(ID)}} \cap Y$ and selects random number $r^{'}, \beta ^{'} \in Z_p$. Then, the algorithm computes:
    $$\begin{aligned} \begin{aligned} {\mathrm{dk}}_{ID,0}&= {\mathrm{sk}}_{{\hat{\delta }}, 0} \cdot g^{r^{'}} = g^{r_{{\hat{\delta }}} + r^{'}}, {\mathrm{dk}}_{{\mathrm{ID}},1} = {\mathrm{uk}}_{{\hat{\delta }},t,1} \cdot g^{\beta ^{'}} = g^{\beta _{{\hat{\delta }}} + \beta ^{'}}, \\ {\mathrm{dk}}_{{\mathrm{ID}},2}&= {\mathrm{sk}}_{{\hat{\delta }},1} \cdot g^{{\mathrm{ar}}^{'}} \cdot {\mathrm{uk}}_{{\hat{\delta }},t,0} \cdot H(t)^{\beta ^{'}} = \mu _{{\hat{\delta }}} \cdot g^{ar_{{\hat{\delta }}}} \cdot \mu _{{\hat{\delta }}}^{-1} \cdot H(t)^{\beta ^{'}} \\&= g^{a(r_{{\hat{\delta }}} + r^{'})} H(t)^{\beta _{{\hat{\delta }}} + \beta ^{'}}, {\mathrm{dk}}_{{\mathrm{ID}},3} = {\mathrm{sk}}_{{\hat{\delta }},2} \cdot g^{r^{'}} = g^{\frac{\alpha + r_{{\mathrm{ID}}}}{a + c}} g^{r_{{\hat{\delta }}} + r^{'}} \end{aligned} \end{aligned}$$
  - For each attribute ${\mathrm{att}}_i \in S$, the algorithm obtains the decryption key components as below:
    $$\begin{aligned} \begin{aligned} {\mathrm{dk}}_{{\mathrm{ID}},i,1} = {\mathrm{sk}}_{i,1}, {\mathrm{dk}}_{{\mathrm{ID}},i,2} = {\mathrm{sk}}_{i,2}, {\mathrm{dk}}_{{\mathrm{ID}},i,3} = {\mathrm{sk}}_{i,3} \end{aligned} \end{aligned}$$
  Finally, the algorithm returns the decryption key for user ID in time period *t* as ${\mathrm{DK}}_{{\mathrm{ID}},t} = \{S, K_{{\mathrm{ID}}}, {\mathrm{dk}}_{{\mathrm{ID}},0}, {\mathrm{dk}}_{{\mathrm{ID}},1}, {\mathrm{dk}}_{{\mathrm{ID}},2}, {\mathrm{dk}}_{{\mathrm{ID}},3}, \{{\mathrm{dk}}_{{\mathrm{ID}},i,1}, {\mathrm{dk}}_{{\mathrm{ID}},i,2}, {\mathrm{dk}}_{{\mathrm{ID}},i,3}\}_{{\mathrm{att}}_i \in S}\}$.

#### Encryption phase

- ${\mathrm{Encrypt}}({\mathrm{PK}}, m, t, {\mathcal {T}}_a) \rightarrow {\mathrm{CT}}_t$: On inputting PK, the data *m* to be encrypted and the designated access policy tree ${\mathcal {T}}_a$, the algorithm consists the following steps for each node $\sigma \in {\mathcal {N}}_t$:
  - The algorithm chooses a random $B \in G_1$ and computes ${\mathrm{ck}} = H_0(B)$ as the symmetric encryption key. Then, it encrypts the data *m* with ck to get ${\mathrm{CT}}_s = {\mathrm{Enc}}(m, ck)$. Moreover, the algorithm computes the message verification code $\phi = H_2(B, m)$.
  - With the designate access policy tree ${\mathcal {T}}_a$ whose root node is denoted by *R*, the algorithm chooses a random number $s_{\sigma ,R}^0 \in Z_p$ as the base secret value of ${\mathcal {T}}_a$ for each node $\sigma \in {\mathcal {N}}_t$ and computes $C_{\sigma ,0} = B \cdot {\hat{e}}(g,g)^{\alpha s_{\sigma ,R}^0}, C_{\sigma ,1} = g^{s_{\sigma ,R}^0}, C_{\sigma ,2} = g^{as_{\sigma ,R}^0}$. Then, for each node *x* in ${\mathcal {T}}_a$, the algorithm picks two random number $s_{\sigma ,x}^1, s_{\sigma ,x}^2 \in Z_p^*$, which satisfy the following equation:
    $$\begin{aligned} {\left\{ \begin{array}{ll} s_{\sigma ,x}^1 \cdot s_{\sigma ,x}^2 = s_{\sigma ,x}^0, &{} x \text { is a time trapdoor}\\ s_{\sigma ,x}^1 = s_{\sigma ,x}^0, s_{\sigma ,x}^2 = 1, &{} \text {otherwise} \end{array}\right. } \end{aligned}$$
  - For each time trapdoor ${\mathrm{TP}}_x$ related to the time release $t \in F_T$ and a secret parameter $s_{\sigma ,x}^2$, DO picks a random number $r_t \in Z_p$ and generates ${\mathrm{TP}}_{\sigma ,x}$ for node $x \in {\mathcal {T}}_a$ as follows:
    $$\begin{aligned} \begin{aligned} {\mathrm{TP}}_{\sigma ,x} = (A_{\sigma ,x} = g^{r_t}, B_x = s_{\sigma ,x}^2 + H_3(e(H_1(t), h)^{r_t})) \end{aligned} \end{aligned}$$
  - For the nodes in access policy tree ${\mathcal {T}}_a$, the algorithm computes the ciphertext in a top-to-bottom way by executing the following steps:
  - For each non-leaf node *x* with $s_{\sigma ,x}^1$, the DO chooses a polynomial $q_x$ whose degree $d_x = th_x - 1$ and $q_x(0) = s_{\sigma ,x}^1$. For each of *x*’s child node $y \in {\mathrm{child}}(x)$ with a unique index ${\mathrm{index}}(y)$, DO set $s_y^0 = q_x({\mathrm{index}}(y))$.
  - For a leaf node *x* with $s_{\sigma ,x}^1$ and related attribute ${\mathrm{att}}_x$, the algorithm generates corresponding ciphertext components $C_{\sigma ,x}, C_{\sigma ,x}^{'}$ as follows:
    $$\begin{aligned} \begin{aligned} \forall x \in X: C_{\sigma ,x} = g^{s_{\sigma ,x}^1}, C_{\sigma ,x}^{'} = H_1({\mathrm{att}}_x)^{s_{\sigma ,x}^1} \end{aligned} \end{aligned}$$
    where *X* is the leaf node set in ${\mathcal {T}}_a$.
  - Moreover, the algorithm obtains $\overrightarrow{C_{\sigma }} = <c_{\sigma ,0}, c_{\sigma ,|b_{\sigma } + 1|}, \ldots , c_{\sigma ,d}>$ by computing:
    $$\begin{aligned} \begin{aligned} c_{\sigma ,0} = (\eta \prod _{i = 1}^{|b_{\sigma }|} \eta _i^{t_i})^{s_{\sigma ,R}^0}, \quad c_{\sigma ,j} = \eta _j^{s_{\sigma ,R}^0} \text { for }j=|b_{\sigma }| + 1\text { to }d \end{aligned} \end{aligned}$$
    If $\sigma$ is a leaf node in ${\mathcal {N}}_t$, $\overrightarrow{C_{\sigma }} = <c_{\sigma ,0}>$, where $c_{\sigma ,0} = H(t)^{s_{\sigma ,R}^0}$.
  Finally, the algorithm outputs the cipertext ${\mathrm{CT}}_t=\{{\mathcal {T}}_a, {\mathrm{CT}}_s, \psi , C_{\sigma ,0}, C_{\sigma ,1}, C_{\sigma ,2}, \{C_{\sigma ,x},C_{\sigma ,x}^{'}\}_{x \in X}, \{{\mathrm{TP}}_{\sigma ,x}\}_{{\mathrm{TP}}_{\sigma ,x} \in {\mathcal {T}}_a}, \overrightarrow{C_{\sigma }}\}_{\sigma \in {\mathcal {N}}_t}$ and uploads it to CSP.
- ${\mathrm{CTUpdate}}({\mathrm{PK}}, {\mathrm{CT}}_t, {\hat{t}}) \rightarrow {\mathrm{CT}}_{{\hat{t}}}$: Given the ciphertext ${\mathrm{CT}}_t$ at time period *t* and a new time period ${\hat{t}}$ that ${\hat{t}} > t$, the algorithm conducts the following steps:
  - For each node $\sigma ^{'} \in {\mathcal {N}}_{{\hat{t}}}$, the algorithm obtains a node $\sigma \in {\mathcal {N}}_t$ which satisfies that $b_{\sigma }$ is a prefix of $b_{\sigma ^{'}}$. Then, the algorithm selects a random number $s_{\sigma ^{'},R}^0 \in Z_p$ as the updated base secret value of ${\mathcal {T}}_a$ and computes:
    $$\begin{aligned} \begin{aligned} C_{\sigma ^{'},0} = C_{\sigma ,0} \cdot {\hat{e}}(g,g)^{\alpha s_{\sigma ^{'},R}^0},C_{\sigma ^{'},1} = C_{\sigma ,1} \cdot g^{s_{\sigma ^{'},R}^0},C_{\sigma ^{'},2} = C_{\sigma ,2} \cdot g^{{\mathrm{as}}_{\sigma ^{'},R}^0} \end{aligned} \end{aligned}$$
    Then, the algorithm computes $s_{\sigma ^{'},x}^1, s_{\sigma ^{'},x}^2$ according to access policy tree ${\mathcal {T}}_a$ the same as in Encrypt algorithm.
  - For each unexposed time trapdoor ${\mathrm{TP}}_x$ related to the new time release ${\hat{t}} \in F_T$ and a secret parameter $s_{\sigma ^{'},x}^2$, DO picks a random number $r_{{\hat{t}}} \in Z_p$ and generates ${\mathrm{TP}}_{\sigma ,x}$ for node $x \in {\mathcal {T}}_a$ as follows:
    $$\begin{aligned} \begin{aligned} {\mathrm{TP}}_{\sigma ^{'}, x} = (A_{\sigma ^{'},x} = A_{\sigma ,x} \cdot g^{r_{{\hat{t}}}}, B_x = s_{\sigma ^{'},x}^2 + H_3(e(H_1({\hat{t}}), h)^{r_{{\hat{t}}}})) \end{aligned} \end{aligned}$$
  - For each leaf node $x \in {\mathcal {T}}_a$, the algorithm calculate the updated ciphertext components as follows:
    $$\begin{aligned} \begin{aligned} \forall x \in X: C_{\sigma ^{'},x} = C_{\sigma ,x} \cdot g^{s_{\sigma ^{'},x}^1},C_{\sigma ^{'},x}^{'} = C_{\sigma ,x}^{'} \cdot H_1({\mathrm{att}}_x)^{s_{\sigma ^{'},x}^1} \end{aligned} \end{aligned}$$
  - Moreover, the algorithm calculates $\overrightarrow{C_{\sigma ^{'}}} = <c_{\sigma ^{'},0}, c_{\sigma ,|b_{\sigma ^{'}} + 1|}, \ldots , c_{\sigma ^{'},d}>$ by computing:
    $$\begin{aligned} \begin{aligned} c_{\sigma ^{'},0} = c_{\sigma ,0} \cdot (\eta \prod _{i = 1}^{|b_{\sigma ^{'}}|} \eta _i^{t_i})^{s_{\sigma ^{'},R}^0} \cdot (\prod _{i = |b_{\sigma }| + 1}^{|b_{\sigma ^{'}}|} c_{\sigma ,i}^{{\hat{t}}_i}) \end{aligned} \end{aligned}$$
    and
    $$\begin{aligned} \begin{aligned} c_{\sigma ^{'},j} = c_{\sigma ,j} \cdot \eta _j^{s_{\sigma ^{'},R}^0} \text { for }j=|b_{\sigma ^{'}}| + 1\text { to} d \end{aligned} \end{aligned}$$
  Finally, the updated ciphertext is generated as ${\mathrm{CT}}_{{\hat{t}}} = \{{\mathcal {T}}_a, {\mathrm{CT}}_s, \psi , C_{\sigma ^{'},0}, C_{\sigma ^{'},1}, C_{\sigma ^{'},2}, \{C_{\sigma ^{'},x},C_{\sigma ^{'},x}^{'}\}_{x \in X}, \{{\mathrm{TP}}_{\sigma ^{'},x}\}_{{\mathrm{TP}}_{\sigma ^{'},x} \in {\mathcal {T}}_a}, \overrightarrow{C_{\sigma ^{'}}}\}_{\sigma ^{'} \in {\mathcal {N}}_{{\hat{t}}}}$ for time period ${\hat{t}}$ and replaces ${\mathrm{CT}}_t$ stored in CSP.

#### Decryption phase

The decryption phase involves the following algorithms:

- ${\mathrm{TokenGen}}({\mathrm{PK}}, t) \rightarrow {\mathrm{TK}}_t$: As the system runs at a uniform time and the time is counted by the number of time point here. When each time point $t \in F_T$ arrives, TA published a time token ${\mathrm{TK}}_t = H_1(t)^{\gamma }$ which can be received by each entity in the system.
- ${\mathrm{Trap}}({\mathrm{PK}}, {\mathrm{TK}}_t) \rightarrow {\mathrm{TP}}_{\sigma ,x}^{'}$: When CSP receives a ${\mathrm{TK}}_t$ at releasing time point *t* published by CA, it finds all trapdoors related to time point *t* in all access policies of files stored in CSP. For each of these trapdoors ${\mathrm{TP}}_{\sigma ,x} = (A_{\sigma ,x}, B_{\sigma ,x})$, the CSP computes the following equation:
  $$\begin{aligned} \begin{aligned} {\mathrm{TP}}_{\sigma ,x}^{'}&= B_{\sigma ,x} - H_2({\hat{e}}({\mathrm{TK}}_t, A_{\sigma ,x})) \\&= s_{\sigma ,x}^2 + H_2(e(H_1(t), g^{\gamma })^{r_t}) - H_2({\hat{e}}(H_1(t)^{\gamma }, g^{r_t})) = s_{\sigma ,x}^2 \end{aligned} \end{aligned}$$
  Then, the CSP replaces these ${\mathrm{TP}}_{\sigma ,x}$ with ${\mathrm{TP}}_{\sigma ,x}^{'}$ for the cipertexts of related files. Thus, if the above equation is correctly executed, the related trapdoor will be exposed to be ${\mathrm{TP}}_{\sigma ,x}^{'} = s_{\sigma ,x}^2$.
- ${\mathrm{TKeyGen}}({\mathrm{PK}}, {\mathrm{DK}}_{{\mathrm{ID}},t}) \rightarrow {\mathrm{TK}}_{{\mathrm{ID}},t}$: On inputting the decryption key ${\mathrm{DK}}_{{\mathrm{ID}},t}$ of DU ID at time period *t*, the algorithm selects $z_t \in _R Z_p^{*}$ as the secret transformation key ${\mathrm{TSK}}_{{\mathrm{ID}}, t}$ of the DU and computes the public transform key ${\mathrm{TPK}}_{{\mathrm{ID}},t} = \{S, K_0, K_1, \{K_{i,j}, K_{i,j}^{'}\}_{{\mathrm{att}}_i \in S}, \{\hat{E_i}^{'}\}_{A_i \in {\mathcal {A}}}\}$, where
  $$\begin{aligned} \begin{aligned} K&= K_{{\mathrm{ID}}}, K_0 = {\mathrm{dk}}_{{\mathrm{ID}},0}^{1/z_t}, K_1 = {\mathrm{dk}}_{{\mathrm{ID}},}^{1/z_t}, K_2 = {\mathrm{dk}}_{{\mathrm{ID}},2}^{1/z_t}, K_3 = {\mathrm{dk}}_{{\mathrm{ID}},3}^{1/z_t},\\&\forall {\mathrm{att}}_i \in S: K_{i,1} = {\mathrm{dk}}_{{\mathrm{ID}},i,1}^{1/z_t}, K_{i,2} = {\mathrm{dk}}_{{\mathrm{ID}},i,2}^{1/z_t}, K_{i,3} = {\mathrm{dk}}_{{\mathrm{ID}},i,3}^{1/z_t} \end{aligned} \end{aligned}$$
  Finally, the algorithm outputs the transformation key pair ${\mathrm{TK}}_{{\mathrm{ID}},t} = ({\mathrm{TPK}}_{{\mathrm{ID}},t}, {\mathrm{TSK}}_{{\mathrm{ID}},t})$ for the DU who keeps the ${\mathrm{TSK}}_u$ secret and publishes ${\mathrm{TPK}}_u$ with data access request.
- ${{\mathrm{Decrypt}}}_{{\mathrm{OUT}}}({\mathrm{PK}}, {\mathrm{TPK}}_{{\mathrm{ID}},t}, {\mathrm{CT}}_t, t) \rightarrow {{\mathrm{CT}}_t}^{'}$: The algorithm is executed by CSP for outsourced decryption given ciphertext ${\mathrm{CT}}_t$. As to each node $x \in {\mathcal {T}}_a$, we assume that the related trapdoor is exposed, i.e. ${\mathrm{TD}}_x^{'} = s_x^2$, as follows:
  $$\begin{aligned} {\left\{ \begin{array}{ll} s_x^2 = {\mathrm{TD}}_x^{'}, &{} x\text { is related to an exposed trapdoor}\\ s_x^2 = 1, &{} x\text { is related to no trapdoor} \end{array}\right. } \end{aligned}$$
  The algorithm picks the ciphertext component corresponding to the leaf node $\sigma _t \in {\mathcal {N}}_t$ and conducts the procedure in a bottom-up way in the following steps:
  - For a leaf node $x \in {\mathcal {T}}_a$, if its associated attribute ${\mathrm{att}}_i \in S$, the algorithm executes:
    $$\begin{aligned} \begin{aligned} P_x&= {\mathrm{DecryptNode}}({\mathrm{CT}}, {\mathrm{TPK}}_u, x) = (\frac{{\hat{e}}(K_{i,1}, C_{\sigma _t,x})}{{\hat{e}}(K_{i,2}^{K} K_{i,3}, C_{\sigma _t,x}^{'})})^{s_{\sigma _t,x}^{2}} \\&= {\hat{e}}(g,g)^{r_{{\mathrm{ID}}} s_{\sigma _t,x}^{0}/z_t} \end{aligned} \end{aligned}$$
    If ${\mathrm{att}}_i \notin S$ or ${\mathrm{TP}}_{\sigma _t, x}$ is not exposed, $P_x = null$.
  - For a non-leaf node $x \in {\mathcal {T}}_a$, If there exists a ${\mathrm{th}}_x$-sized child nodes set ${\mathcal {N}}_x$ of *x*, for each node $y \in {\mathcal {N}}_x$, $P_x \ne {\mathrm{null}}$ and there exists no unexposed trapdoor associated with node *x* at time period *t*. The algorithm executes the following equation:
    $$\begin{aligned} \begin{aligned} P_x&= {\mathrm{DecryptNode}}({\mathrm{CT}}, {\mathrm{TPK}}_u, x) = (\prod _{y \in {\mathcal {N}}_x} P_y^{\bigtriangleup _{j, {\mathcal {N}}_x^{'}}})^{s_{\sigma _t,x}^{2}} \\&= {\hat{e}}(g,g)^{r_{{\mathrm{ID}}} s_{\sigma _t,x}^{0}/z_t} \end{aligned} \end{aligned}$$
    where $j = {\mathrm{index}}(y)$ and ${\mathcal {N}}_x^{'} = \{index(y): y \in {\mathcal {N}}_x\}$. Otherwise, $P_x = {\mathrm{null}}$. Then, recursively, the algorithm obtains $P_R = {\hat{e}}(g,g)^{r_{{\mathrm{ID}}} s_{\sigma _t,R}^{0}/z_t}$ for root node *R* of ${\mathcal {T}}_a$.
  $$\begin{aligned} \begin{aligned} C_{\sigma _t}^{'} = \frac{{\hat{e}}(K_3, C_{\sigma _t, 1}^{K} C_{\sigma _t, 2}) \cdot {\hat{e}}(K_1, c_{\sigma _t, 0})}{{\hat{e}}(K_0^{K} K_2, C_{\sigma _t, 1}) \cdot P_R} = {\hat{e}}(g,g)^{\alpha s_{\sigma _t,x}^{0}/z_t} \end{aligned} \end{aligned}$$
  Finally, the CSP sends partially decrypted ciphertext ${\mathrm{CT}}_{\sigma _t}^{'} = \{C_{\sigma _t}^{'}, {\mathrm{CT}}_s, \psi , C_{\sigma _t, 0}\}$ to the DU.
- ${{\mathrm{Decrypt}}}_U ({\mathrm{PK}}, {\mathrm{TSK}}_{{\mathrm{ID}},t}, {\mathrm{CT}}_{\sigma _t}^{'}) \rightarrow \text {M or null}$: After receiving the partially decrypted ciphertext ${\mathrm{CT}}_{\sigma _t}^{'}$, the algorithm gets the random element $B^*$ by computing $B^* = C_{\sigma _t, 0}/(C_{\sigma _t}^{'})^{{\mathrm{TSK}}_{{\mathrm{ID}},t}}$. Then, it calculates ${\mathrm{ck}}^* = H_0(B^*), m^* = {\mathrm{Dec}}({\mathrm{CT}}_s, {\mathrm{ck}}^{*})$. It outputs $m^*$ if $\psi = H_2(B^*, m^*)$ or null otherwise.

#### User trace phase

- ${\mathrm{UTrace}}({\mathrm{PK}}, {\mathrm{DK}}_{{\mathrm{ID}},t}, {\mathrm{RL}}_u, t) \rightarrow {\mathrm{ID}} \text { or } null$: Given a suspected decryption key ${\mathrm{DK}}_{{\mathrm{ID}},t}$, the algorithm first runs the following check. **Key Sanity Check**:
  1 $$\begin{aligned}&K_{{\mathrm{ID}}} \in Z_p, {\mathrm{dk}}_{{\mathrm{ID}},0}, {\mathrm{dk}}_{{\mathrm{ID}},1}, {\mathrm{dk}}_{{\mathrm{ID}},2}, {\mathrm{dk}}_{{\mathrm{ID}},3}, {\mathrm{dk}}_{{\mathrm{ID}},i,1}, k_{{\mathrm{ID}},i,2}, {\mathrm{dk}}_{{\mathrm{ID}},i,3} \in G_1 \end{aligned}$$
  2 $$\begin{aligned}&{\hat{e}}(g, {\mathrm{dk}}_{{\mathrm{ID}},2}) = {\hat{e}}(g^a, {\mathrm{dk}}_{{\mathrm{ID}},0}) {\hat{e}}(H(t), {\mathrm{dk}}_{{\mathrm{ID}},1}) \nonumber \\&\quad \exists {\mathrm{att}}_i \in S, \text { s.t } {\hat{e}}({\mathrm{dk}}_{{\mathrm{ID}},3}, g^a \cdot g^{K_{{\mathrm{ID}}}}) \cdot {\hat{e}}({\mathrm{dk}}_{{\mathrm{ID}},1}, H(t)) \cdot \end{aligned}$$
  3 $$\begin{aligned}&{\hat{e}}({\mathrm{dk}}_{{\mathrm{ID}},i,2}^{K_{{\mathrm{ID}}}}{\mathrm{dk}}_{{\mathrm{ID}},i,3},H_1({\mathrm{att}}_i)) = {\hat{e}}({\mathrm{dk}}_{{\mathrm{ID}},i,1}, g) \cdot \nonumber \\&\quad {\hat{e}}({\mathrm{dk}}_{{\mathrm{ID}},0}^{K_{{\mathrm{ID}}}}{{\mathrm{dk}}_{{\mathrm{ID}}},2}, g) \cdot {\hat{e}}(g,g)^{\alpha } \end{aligned}$$
  If the decryption key ${\mathrm{DK}}_{{\mathrm{ID}},t}$ does not satisfy **Key Sanity Check**, the algorithm abort and outputs null. Otherwise, we consider it as a well-formed decryption key. Then, the algorithm computes ${\mathrm{ID}} = {\mathrm{Dec}}(K_{{\mathrm{ID}}}, {\hat{K}})$ and recovers the suspected user ID. The algorithm checks whether ID is stored in $\mathcal {BT}$. If ID does node exists, the algorithm aborts and returns *null*. Otherwise, it updates the revocation list with ${\mathrm{RL}}_u = {\mathrm{RL}}_u \cup \{({\mathrm{ID}},t)\}$.

## Results and discussion

In this section, we present the result of our study including security and performance analysis with corresponding discussion.

### Security analysis

#### Traceability of TR-TABE

In this part, we show the reduction of the traceability of our TR-TABE scheme to *l*-SDH assumption.

##### *Theorem 1*

*Our TR-TABE scheme is traceable if* *l*-*SDH assumption holds and the number of queries *$q < l$.

##### *Proof*

If there exists a PPT adversary ${\mathcal {A}}$ that can break the traceability game with non-negligible advantage $\varepsilon$ after *q* queries, then we can construct a challenger ${\mathcal {C}}$ that have the ability to solve the *l*-SDH problem with advantage $\varepsilon /2$ assuming $l = q + 1$. We also suppose $G_0, G_1$ are two bilinear groups of prime order *p* with a bilinear map ${\hat{e}}: G_0 \times G_0 \rightarrow G_1$ and a generator $h \in G_0$. After receiving a *l*-SDH problem input $({\hat{g}}, {\hat{g}}^{a}, {\hat{g}}^{a^2}, \ldots , {\hat{g}}^{a^l})$, ${\mathcal {C}}$ sets $T_i = {\hat{g}}^{a^i}$ where $i \in \{0, \ldots , l\}$ and intends to output $(c_t, w_t = {\hat{g}}^{1/(a + c_0)})$. ${\mathcal {C}}$ creates the following simulation in utilizing ${\mathcal {A}}$ to break the scheme by solving the *l*-SDH problem. $\square$

*Init*. ${\mathcal {C}}$ runs *Setup* to initiate the whole system and randomly selects *q* numbers $c_1, c_2, \ldots , c_q \in Z_p$ and $\alpha , \gamma \in _R Z_p, \{\eta _i\}_{i \in [d]} \in _R G_0$. Suppose a polynomial $q(x) = \prod _{i=1}^{q} (x + c_i) = \sum _{i=0}^{q} \zeta _i x^i$, where $\zeta _0, \ldots , \zeta _q \in Z_p$. ${\mathcal {C}}$ sets $g = \prod \nolimits _{i=0}^{q} (T_i)^{\zeta _i} = {\hat{g}}^{q(a)}$ and $g^a = \prod \nolimits _{i=1}^{q+1} (T_i)^{\zeta _{i-1}} = {\hat{g}}^{q(a) \cdot a}$. ${\mathcal {C}}$ publishes system public key ${\mathrm{PK}}=\{G_0, G_1, {\hat{e}}, g, g^a, {\hat{e}}(g,g)^{\alpha }, \eta _1, \ldots , \eta _d, h = g^{\gamma }\}$.

*Key query*. ${\mathcal {A}}$ submits $({\mathrm{ID}}_i, S_i)$ to ${\mathcal {C}}$ for the corresponding decryption key ${\mathrm{DK}}_{{\mathrm{ID}},t}$ at time point *t*. For the *i*th query that $i \le q$, suppose $q_i(x) = q(x)/(x + c_i) = \prod \nolimits _{j=1, j \ne i}^{q} (x+c_j) = \sum _{j=0}^{q-1} \zeta _j x^j$. ${\mathcal {C}}$ computes $\tau _i = \prod \nolimits _{j=0}^{q} (T_j)^{\zeta _j}$ = ${\hat{g}}^{q_i(a)} = {\hat{g}}^{q(a)/(a+c_i)} = g^{1/(a+c_i)}$. ${\mathcal {C}}$ randomly chooses $r_{\delta }, r_{{\mathrm{ID}}_i}, \beta _{\delta } \in Z_p$, $\{r_y\}_{{\mathrm{att}}_y \in S_i} \in Z_p$ then computes the decryption key by simulating AKeyGen, UKeyGen and DKeyGen,

$$\begin{aligned} \begin{aligned} K_{{\mathrm{ID}}_i}&= c_i, {\mathrm{dk}}_{{\mathrm{ID}}_i,0} = g^{r_{\delta }}, {\mathrm{dk}}_{{\mathrm{ID}}_i,1} = g^{\beta _{\delta }}, {\mathrm{dk}}_{{\mathrm{ID}}_i,2} = g^{a r_{\delta }} H(t)^{\beta _{\delta }}, {\mathrm{dk}}_{{\mathrm{ID}}_i,3} = g^{\frac{\alpha + r_{{\mathrm{ID}}_i}}{a + c_i}} g^{r_{\delta }} \\ \{{\mathrm{dk}}_{{\mathrm{ID}},y,1}&= g^{r_{{\mathrm{ID}}_i}} H_1({\mathrm{att}}_y)^{r_y}, {\mathrm{dk}}_{{\mathrm{ID}}_i,y,2} = g^{r_y}, {\mathrm{dk}}_{{\mathrm{ID}}_i,y,3} = g^{{\mathrm{ar}}_y}\}_{{\mathrm{att}}_y \in S_i} \end{aligned} \end{aligned}$$

Finally, ${\mathcal {C}}$ outputs ${\mathrm{DK}}_{{\mathrm{ID}}_i,t} = \{S_i, K_{{\mathrm{ID}}_i}, {\mathrm{dk}}_{{\mathrm{ID}}_i,0}, {\mathrm{dk}}_{{\mathrm{ID}}_i,1}, {\mathrm{dk}}_{{\mathrm{ID}}_i,2}, {\mathrm{dk}}_{{\mathrm{ID}}_i,3}, \{{\mathrm{dk}}_{{\mathrm{ID}}_i,y,1}, {\mathrm{dk}}_{{\mathrm{ID}}_i,y,2}, {\mathrm{dk}}_{{\mathrm{ID}}_i,y,3}\}_{{\mathrm{att}}_y \in S_i}\}$ to ${\mathcal {A}}$.

*Key Forgery*. ${\mathcal {A}}$ submits a decryption key ${\mathrm{DK}}_{{\mathrm{ID}}_i,t}^*$ to ${\mathcal {C}}$. Suppose an event that ${\mathcal {A}}$ breaks the game is denoted by $E_{{\mathcal {A}}}$ in which ${\mathrm{DK}}_{{\mathrm{ID}}_i,t}^*$ can pass **Key Sanity Check** of Eqs. 1, 2, 3 and $K_{{\mathrm{ID}}_i} \notin \{c_1, \ldots , c_q\}$. In case that $E_{{\mathcal {A}}}$ does not happen, ${\mathcal {C}}$ randomly chooses $(c_{{\hat{t}}}, w_{{\hat{t}}}) \in Z_p \times G_0$ as a response to *l*-SDH problem. Otherwise, ${\mathcal {C}}$ represents $q(x) = \vartheta (x)(x + K_{{\mathrm{ID}}_i}) + \vartheta -1$, where $\vartheta (x) = \sum _{i=0}^{q-1}\vartheta _i x^i$ and $\vartheta - 1 \ne 0$. Thus, $q(x) \text { mod } (x + K_{{\mathrm{ID}}_i}) \ne 0$ as $q(x) = \prod \nolimits _{i=1}^{q}(x + c_i)$ and $K_{{\mathrm{ID}}_i} \notin \{c_1, \ldots c_q\}$. Then, ${\mathcal {C}}$ can calculate $1/(\vartheta -1)$ as ${\mathrm{gcd}}(\vartheta -1,p)=1$. It also computes $\tau = (\frac{{\mathrm{dk}}_{{\mathrm{ID}}_i,3}}{{\mathrm{dk}}_{{\mathrm{ID}}_i,1}})^{(\alpha + r_{{\mathrm{ID}}_i})^{-1}} = g^{\frac{1}{a+K_{{\mathrm{ID}}_i}}} = {\hat{g}}^{\frac{q(a)}{a+K_{{\mathrm{ID}}_i}}} = {\hat{g}}^{\vartheta (a)}{\hat{g}}^{\frac{\vartheta - 1}{a+K_{{\mathrm{ID}}_i}}}, w_{{\hat{t}}} = (\tau \prod \nolimits _{i=0}^{q-1} T_i^{-\vartheta _i})^{\frac{1}{\vartheta - 1}} = {\hat{g}}^{\frac{1}{a+K_{{\mathrm{ID}}_i}}}, c_{{\hat{t}}} = K_{{\mathrm{ID}}_i} (\text {mod } p)$. Then, ${\hat{e}}({\hat{g}}^{c_{{\hat{t}}}} \cdot {\hat{g}}^{w_{{\hat{t}}}}, {\hat{g}}^{\frac{1}{a+K_{{\mathrm{ID}}_i}}}) = {\hat{e}}({\hat{g}}, {\hat{g}})$ and $(c_{{\hat{t}}}, w_{{\hat{t}}})$ is a solution for *l*-SDH problem.

Suppose another event $E_{{\mathrm{SDH}}}(c_t, w_t)$ that $(c_t, w_t)$ can solve *l*-SDH problem which satisfies ${\hat{e}}({\hat{g}}^{c_{t}} \cdot {\hat{g}}^{w_{t}}, {\hat{g}}^{\frac{1}{a+K_{{\mathrm{ID}}_i}}}) = {\hat{e}}({\hat{g}}, {\hat{g}})$. The event $E_{{\mathrm{SDH}}}(c_t, w_t)$ happens if and only if $E_{{\mathcal {A}}}$ happens and ${\mathrm{gcd}}(\vartheta -1,p)=1$ given $(c_t, w_t)$ from ${\mathcal {C}}$. Therefore, the probability that ${\mathcal {C}}$ solves *l*-SDH problem is

$$\begin{aligned} \begin{aligned} {\mathrm{Pr}}[E_{{\mathrm{SDH}}}(c_t, w_t)]&= {\mathrm{Pr}}[E_{{\mathrm{SDH}}}(c_t, w_t)|\overline{E_{{\mathcal {A}}}}] \cdot {\mathrm{Pr}}[\overline{E_{{\mathcal {A}}}}] \\&\quad + {\mathrm{Pr}}[E_{{\mathrm{SDH}}}(c_t, w_t)|E_{{\mathcal {A}}} \wedge {\mathrm{gcd}}(\vartheta -1,p)\ne 1] \cdot {\mathrm{Pr}}[E_{{\mathcal {A}}} \wedge {\mathrm{gcd}}(\vartheta -1,p)\ne 1] \\&\quad + {\mathrm{Pr}}[E_{{\mathrm{SDH}}}(c_t, w_t)|E_{{\mathcal {A}}} \wedge {\mathrm{gcd}}(\vartheta -1,p)= 1] \cdot {\mathrm{Pr}}[E_{{\mathcal {A}}} \wedge {\mathrm{gcd}}(\vartheta -1,p)= 1] \\&= 0 + 0 + 1 \cdot {\mathrm{Pr}}[E_{{\mathcal {A}}} \wedge {\mathrm{gcd}}(\vartheta -1,p)= 1] \\&= {\mathrm{Pr}}[E_{{\mathcal {A}}} \wedge {\mathrm{gcd}}(\vartheta -1,p)= 1] \\&= {\mathrm{Pr}}[E_{{\mathcal {A}}}] \cdot {\mathrm{Pr}}[{\mathrm{gcd}}(\vartheta -1,p)= 1] = {\mathrm{Pr}}[E_{{\mathcal {A}}}] \end{aligned} \end{aligned}$$

As the probability of $E_{{\mathrm{SDH}}}(c_t, w_t)$ with random $(c_t, w_t)$ is negligible, we set it as zero in our computation.

In conclusion, if ${\mathcal {A}}$ can break our scheme with non-negligible advantage, then ${\mathcal {C}}$ can solve *l*-SDH problem with same advantage, which is inconsistent with *l*-SDH assumption. Therefore, our scheme is traceable.

#### IND-CPA security of TR-TABE

##### **Theorem 2**

*No PPT adversaries can selectively win the security game of our scheme with an advantage that is non-negligible on condition the DBDH assumption holds.*

##### *Proof*

When the advantage $\varsigma$ of adversary ${\mathscr {A}}$ is non-negligible when he selectively break the security game against our scheme, we can create a simulator ${\mathscr {B}}$ who is able to distinguish a DBDH parameter from a random parameter with an identical advantage to that of ${\mathcal {A}}$. $\square$

*Init*: The simulator ${\mathscr {B}}$ of DBDH game creates the bilinear group $\{G_0, G_1, {\hat{e}}, p, g\}$, where ${\hat{e}}: G_0 \times G_0 \rightarrow G_1$ and $g \in G_0$. It than selects random $c,d,m,\nu \in Z_p$ and $\varepsilon \in [0,1]$. If $\varepsilon =0$, the challenger ${\mathscr {B}}$ generates a tuple $(C,D,M,V)=(g^c, g^d, g^m, {\hat{e}}(g,g)^{{\mathrm{cdm}}})$; otherwise, it generates $(g^c, g^d, g^m, {\hat{e}}(g,g)^{\nu })$. ${\mathscr {B}}$ then sends the tuple to ${\mathscr {C}}$. In the meantime, the adversary ${\mathscr {A}}$ submits a selected challenging access policy tree ${\mathcal {T}}_a^*$, a time period $t^* \in F_T$ and a revocation list ${\mathrm{RL}}_u^*$ which contains revoked users before the time period to challenger ${\mathscr {C}}$ of our scheme.

*Setup*: After the challenger ${\mathscr {C}}$ gets the DBDH tuple (*C*, *D*, *M*, *V*) and bilinear group from ${\mathscr {B}}$ as well as the security parameters $N_u, \lambda , d$, it computes $N_t = 2^d$ as the total number of time periods and randomly chooses $\alpha ^{'}, \gamma \in Z_p$, sets ${\hat{e}}(g,g)^{\alpha } = {\hat{e}}(g,g)^{\alpha ^{'}} {\hat{e}}(g,g)^{cd}$. and hash functions $H_0, H_1, H_2, H_3$. Then, for Type-*A* attack, ${\mathscr {C}}$ computes $\delta = g^{\gamma }$ and for Type-*B* attack, it simulates $\delta = D$. ${\mathscr {C}}$ also simulates $H_1(x) = g^{q_x}$, where $q_x \in Z_p$. Finally, ${\mathscr {C}}$ generates system public parameters ${\mathrm{PK}}=\{G_0,G_1,p, g, g^{a}, {\hat{e}}, {\hat{e}}(g,g)^{\alpha }, \eta _1, \ldots , \eta _d, h = g^{\gamma }\}$ and the master key ${\mathrm{MSK}} = \{g^{\alpha }, a, \gamma \}$. It keeps the MSK privately and sends the PK to the adversary ${\mathscr {A}}$.

*Phase 1*: The adversary ${\mathscr {A}}$ submits a series queries $q_i$ for secret key and transformation key as follows:

- *SK Query*: ${\mathscr {C}}$ generates secret attribute key given the attribute set *S* in ${\mathscr {A}}$’s requests. It first sets random $\mu _{\delta }^{'} \in G_0$ for each node $\delta \in \mathcal {BT}$.
  - Type-*A* adversary: ${\mathcal {T}}_a^{*}(S) \ne 1$. To generate the secret attribute key, ${\mathscr {C}}$ chooses an empty leaf node $\theta \in \mathcal {BT}$ randomly if the identity ID is not assigned. It picks random numbers $r_{\delta }, c^{'}, r^{'} \in Z_p$ and sets $r_{{\mathrm{ID}}} = r^{'} - c$.
    $$\begin{aligned} \begin{aligned} K_{{\mathrm{ID}}}&= c^{'}, {\mathrm{sk}}_{\delta , 0} = g^{r_{\delta }}, {\mathrm{sk}}_{\delta , 1} = \mu _{\delta }^{'} \cdot g^{{\mathrm{ar}}_{\delta }}, {\mathrm{sk}}_{\delta , 2} = g^{\frac{\alpha + r^{'} - c}{a + c^{'}}} g^{r_{\delta }}, \\&\{{\mathrm{sk}}_{i,1} = g^{r_{{\mathrm{ID}}}} H_1({\mathrm{att}}_i)^{r_i} = \frac{g^{r^{'}}H_1({\mathrm{att}}_i)^{r_i}}{C}, {\mathrm{sk}}_{i,2} = g^{r_i}, {\mathrm{sk}}_{i,3} = g^{{\mathrm{ar}}_i}\}_{{\mathrm{att}}_i \in S} \end{aligned} \end{aligned}$$
  - Type-*B* adversary: ${\mathcal {T}}_a^*(S) = 1$ and ${\mathrm{ID}} \in {\mathrm{RL}}_u^*$. Then, ID must have been associated with a leaf node $\theta \in \mathcal {BT}$ and each node $\delta \in {\mathrm{Path(ID)}}$ is also assigned. ${\mathscr {C}}$ returns the original secret key to ${\mathscr {A}}$.
- *UK Query*: ${\mathscr {C}}$ creates update key on inputting a revocation list ${\mathrm{RL}}_u$ and a time period *t*. For each node $\delta \in Y$, ${\mathscr {C}}$ chooses a random number $\beta _{\delta } \in Z_p$ for each node $\delta$ and computes: ${\mathrm{UK}}_{\delta ,t} = \{{\mathrm{uk}}_{\delta , t,0} = \mu _{\delta }^{-1} \cdot H(t)^{\beta _{\delta }}, {\mathrm{uk}}_{\delta , t, 1} = g^{{\beta _{\delta }}}\}$
- *DK Query*: ${\mathscr {C}}$ queries this algorithm to generate the final decryption key as follows.
  $${\mathrm{dk}}_{{\mathrm{ID}},0} = g^{r_{\delta }}, {\mathrm{dk}}_{{\mathrm{ID}},1} = g^{\beta _{\delta }}, {\mathrm{dk}}_{{\mathrm{ID}},2} = g^{ar_{\delta }} H(t)^{\beta _{\delta }}, {\mathrm{dk}}_{{\mathrm{ID}},3} = g^{\frac{\alpha + r^{'} - c}{a + c^{'}}} g^{r_{\delta }}$$
- *TK Query*: Similar to the *SK Query*, the challenger ${\mathscr {C}}$ runs TKeyGen algorithm to generate transformation key pair and sends them to ${\mathscr {A}}$.
- *RL Query*: Given the query of ${\mathscr {A}}$ for user revocation request with identity ID at time period *t*, ${\mathscr {C}}$ updates the revocation list by adding $({\mathrm{ID}}, t)$ into revocation list ${\mathrm{RL}}_u$.

*Challenge*: The adversary ${\mathscr {A}}$ finishes the *Phase 1* and submits two data $B_0$ and $B_1$ with equal length to ${\mathscr {C}}$. First, ${\mathscr {C}}$ picks $\epsilon \in _R [0,1]$ and computes $C_{\sigma ,0} = B_{\epsilon } \cdot {\hat{e}}(g,g)^{\alpha s_{\sigma ,R}^0} = B_{\epsilon } \cdot {\hat{e}}(g,g)^{\alpha m} = B_{\epsilon } \cdot V{\hat{e}}(g,g)^{\alpha ^{'} m}, C_{\sigma ,1} = g^{s_{\sigma ,R}^0} = g^m = M, C_{\sigma ,2} = g^{{\mathrm{as}}_{\sigma ,R}^0} = g^{{\mathrm{am}}} = M^a$.

With respect to the adversary ${\mathscr {A}}$, when $\varepsilon = 0$, $V={\hat{e}}(g,g)^{{\mathrm{cdm}}}$ and according to the decryption procedure, the adversary can get $B_{\epsilon }$ from CT. Nevertheless, when $\varepsilon =1$, $V \in G_1$ is a random element. Thus, ${\mathscr {A}}$ cannot get any information about $m_{\epsilon }$ from CT.

*Phase 2*: The adversary ${\mathscr {A}}$ repeats the procedures in *Phase 1* with the same restriction that the ${\mathrm{ID}} \notin {\mathrm{RL}}$ and the attribute set ${\mathcal {S}}$ in queries do not satisfy *T*.

*Guess*: The adversary ${\mathscr {A}}$ outputs the guess of bit $\epsilon ^{'}$. If $\epsilon = \epsilon ^{'}$, the challenger ${\mathscr {C}}$ guesses $V = {\hat{e}}(g,g)^{{\mathrm{cdm}}}$ with his output 0; otherwise, it guesses *Z* as a random element. If the adversary ${\mathscr {A}}$ has the advantage of $\varsigma$, then the challenger ${\mathscr {C}}$ can break the DBDH game with advantage $\frac{\varsigma }{2}$ given that the variables $\epsilon$ and $\varepsilon$ are independent. The computation of the advantage for ${\mathscr {C}}$ is the same as in [12].

In conclusion, if an adversary ${\mathscr {A}}$ can win the security game of our scheme with a non-negligible advantage $\varsigma$, then the challenger ${\mathscr {C}}$ can break the DBDH game with identical advantage. Therefore, our scheme is IND-CPA secure in our security model.

### Performance analysis

In this section, we compare our proposal and several existing related work in theoretical analysis and actual performance evaluation. We first present the function comparison of our scheme and various state-of-the-art CP-ABE schemes [6, 12, 21, 46, 48] summarized in Table 1.

**Table 1:** Function comparison

Scheme	User traceability	User revocability	Backward security	Forward security	Time-based access control	Efficient decryption
Scheme [12]	$\times$	$\times$	$\times$	$\times$	$\checkmark$	$\times$
Scheme [48]	$\checkmark$	$\times$	$\times$	$\times$	$\times$	$\times$
Scheme [46]	$\checkmark$	$\checkmark$	$\checkmark$	$\times$	$\times$	$\times$
Scheme [6]	$\times$	$\checkmark$	$\checkmark$	$\checkmark$	$\times$	$\times$
Scheme [21]	$\times$	$\checkmark$	$\checkmark$	$\times$	$\checkmark$	$\checkmark$
Our scheme	$\checkmark$	$\checkmark$	$\checkmark$	$\checkmark$	$\checkmark$	$\checkmark$

#### Theoretical analysis

The theoretical analysis of our scheme involves computation and storage complexity. For computation complexity, we let $|X|, K, N, |{\mathrm{TP}}|,|P|$ denote the size of leaf node set, non-leaf node set, node set of ${\mathcal {N}}_t$, trapdoors set and the length of the path in binary tree $\mathcal {BT}$ in access policy tree ${\mathcal {T}}_a$. Then, we let $E_0, M_0$ and $E_1, M_1$ denote the exponential operation and the multiplication operation in $G_0$ and $G_1$, respectively while let *P* denote the pairing operations. For storage complexity, we let $|G_0|, |G_1|$ and $|Z_p|$ denote the length of elements in $G_0, G_1$ and $Z_p$, respectively. In Table 2, we compare the TR-TABE with scheme [12] from the aspect of computation cost of Encrypt, Decrypt, KeyGen and storage overhead involving public parameter size (PP Size), decryption key size (DSK Size) and ciphertext size (CT Size). Here, the KeyGen denotes the AKeyGen algorithm in TR-TABE and DSK Size in scheme [12] is the size of user secret key.

**Table 2:** Theoretical performance comparison

Scheme	Encrypt	Decrypt	KeyGen
Scheme [12]	$(2|X| + |{\mathrm{TP}}| + 1)E_0$ $+ (|{\mathrm{TP}}| + 1)E_1$ $+ M_1 + |{\mathrm{TP}}|P$	$(|X| + K)E_1 +$ $(2|X| + K + 2)M_1$ $+ (2|X| + 1)P$	$(3|S|+1)E_0$ $+ |S|M_0$
Our scheme	$N((2|X|+d+|{\mathrm{TP}}|$ $+2)E_0+(|{\mathrm{TP}}|+1)E_1$ $+ M_1+|{\mathrm{TP}}|P)$	$E_1 + M_1$	$(4|S| + 3|P|)E_0$ $+ (|S| + 2|P|)M_0$
Scheme	PP size	DSK size	CT size
Scheme [12]	$3|G_0|+|G_1|$	$(2|S|+1)|G_0|$	$(2|X|+|{\mathrm{TP}}|+1)|G_0|$ $+ |G_1| + |{\mathrm{TP}}||Z_p|$
Our scheme	$(d+2)|G_0| + |G_1|$	$|Z_p|$	$|N|((2|X|+|{\mathrm{TP}}|+3)|G_0|$ $+ |G_1| + |{\mathrm{TP}}||Z_p|)$

#### Implementation and evaluation

As for the precise performance evaluation for our scheme, we implement it and the related scheme [12] using Java Programming Language and Java Pairing-Based Cryptography library (JPBC) [49] which supports operations of pairing, exponential, addition, multiplication and inversion in finite field and groups. In our implementation, we adopt the Type-A curve with prime order. It is defined over a 160-bit elliptic curve group and a 512-bit finite field. Moreover, our experimental simulations are run on a Windows10 system with Intel Core i5 CPU 2.13 GHz and 8.00-GB RAM.

**Fig. 4:**
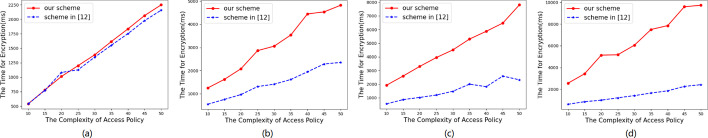
Encryption cost comparison **a** $|N|= 1$, **b** $|N|= 2$, **c** $|N|= 3$ and **d** $|N|= 4$

Figure 4 shows the actual time cost comparison of *Encrypt* algorithm in different settings. The time cost of encryption in our scheme is affected by the number of leaf nodes, the number of trapdoors in access policy tree ${\mathcal {T}}_a$ and the size of ${\mathcal {N}}_t$ while that of [12] is only affected by the former two. However, we can see from Fig. 4a–d that the time cost difference between the two schemes is increasing with the growth of |*N*|.

**Fig. 5:**
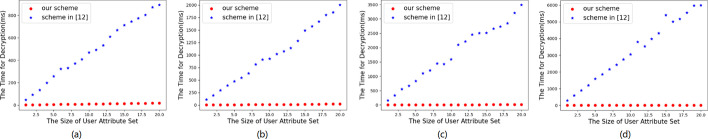
Decryption cost comparison **a** $|S| = 2$, **b** $|S| = 5$, **c** $|S| = 10$ and **d** $|S| = 20$

In Fig. 5, we notice that within the same size of user attribute set, the decryption cost in our scheme is smaller and nearly constant when the number of ciphertexts grows, while in scheme [12], it is far more and linear with the growth of the number of ciphertexts. Moreover, from Fig. 5a–d, within the same size of user attribute set, the time cost of decryption for one data file in scheme [12] is far more than that of our scheme, and as the number of ciphertexts grows, the gap gets bigger.

**Fig. 6:**
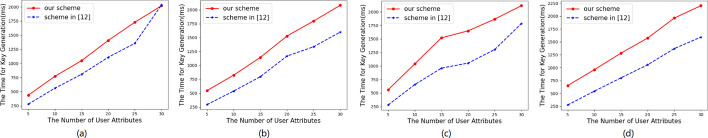
Key Generation cost comparison **a** $|P| = 4$, **b** $|P| = 6$, **c** $|P| = 8$, **d** $|P| = 10$

Figure 6 depicts the actual time cost of key generation in the two schemes. From the theoretical analysis, the time cost of key generation in our scheme is affected by the number of attributes of a user and the length of the user path in $\mathcal {BT}$ of the system, which incurs extra time cost. As Fig. 6a–d shows, when |*P*| is set as [4, 6, 8, 10], the time cost in our scheme is more than that of scheme [12] and the difference of time cost between the two schemes increases with the growth of |*P*|. This is consistent with the theoretical analysis of our scheme which incurs the extra cost for key components corresponding to the path for the users in $\mathcal {BT}$. These extra components are used for user revocation with forward security. As the user secret attribute key is generated by TA just once for each user, the extra cost is also acceptable for DU.

**Fig. 7:**
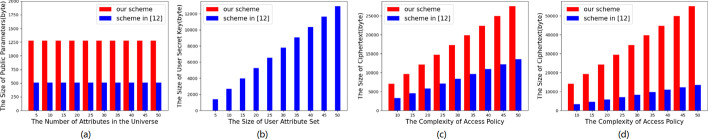
Storage overhead comparison **a** Public Parameter Size, **b** Decryption Key Size, Ciphertext Size: **c** $|N|=2$ and **d** $|N|=4$

Figure 7 plots the comparison of the size of public parameter, decryption key and ciphertext between the two schemes. Obviously, in Fig. 7a, the size of the system public parameters in our scheme is related to *d* (Here, we set $d=6$) and is larger than that of scheme [12] as we take some extra storage complexity for time function *H* in our scheme. Moreover, in Fig. 7b, the storage overhead of decryption key in our scheme is nearly none due to outsourced decryption, while that of the scheme [12] is far more and proportional to the size of the user attribute set. From Fig. 7c, d with different settings of |*N*|, we notice that the ciphertext size in our scheme is more than that of [12] and as |*N*| grows, the gap becomes bigger.

**Table 3:** User tracing complexity comparison

Scheme	Computation cost	Storage overhead
Scheme in [47]	*O*(*N*)	$n|Z_N|$
Our scheme	$O(1) + {\mathrm{Dec}}$	−

**Fig. 8:**
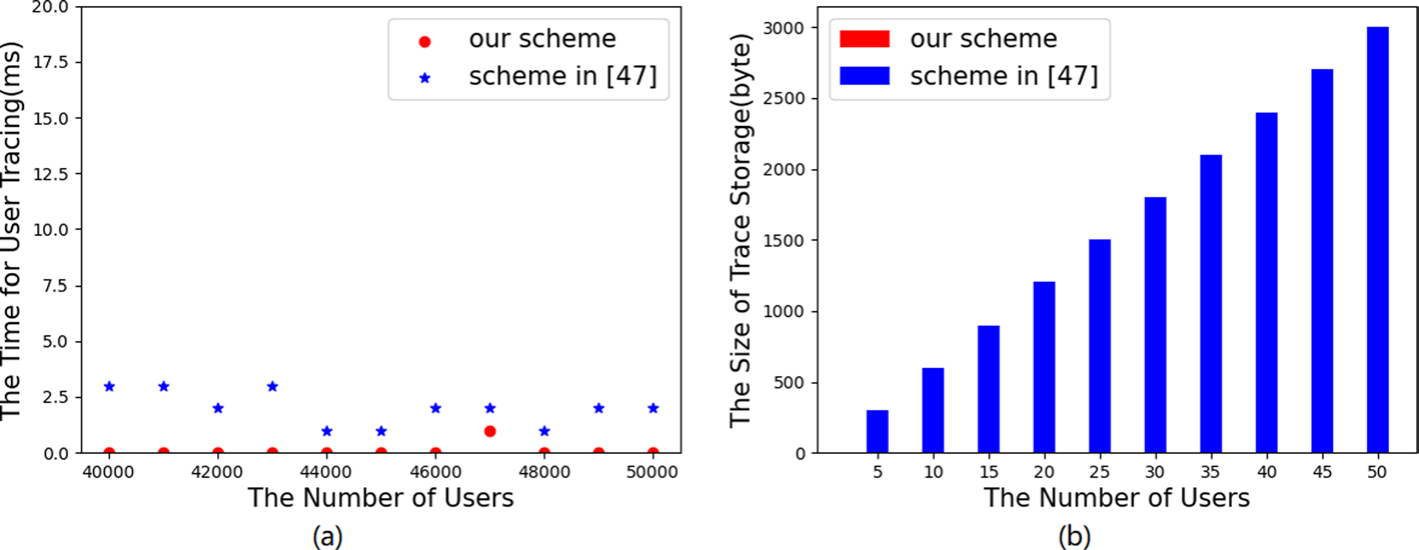
User tracing cost comparison **a** Time cost **b** Storage overhead

In Fig. 8, we present the comparison of the actual time and storage cost in user tracing between our scheme and the scheme [47]. Accordingly, we summarize the theoretical overhead comparison of the two schemes in Table 3. The user tracing algorithm in our scheme eliminates the identity table, which only costs *O*(1) in computational complexity and no storage overhead, while the scheme [47] costs *O*(*n*) in computation complexity for user tracing, where *n* is the size of identity table, and $n|Z_N|$ in storage, where *N* is a composite number. We observe in Fig. 8a and b that the time cost and storage overhead in user tracing of our scheme is nearly none.

In conclusion, although our scheme incurs extra storage overhead for ciphertext, it outperforms existing related schemes in time cost of encryption and decryption as well as user tracing. Moreover, it can support large attribute universe, user traceability and revocability with forward and backward security and time-based data access control. Thus, our scheme is more suitable for data sharing of smart city applications with resource-poor user devices.

## Conclusion

In this paper, we investigated the troublesome security issues of data sharing in cloud-fog-based smart city and proposed an efficient time-based data sharing scheme with traceability and revocability, i.e., TR-TABE. We presented the system model and the threat model for our proposal. To support efficient user tracing and revocation, we designed an efficient traceable and revocable CP-ABE scheme with forward and backward security. Moreover, to provide time-based data access control, we integrated the time release encryption into our designed CP-ABE scheme. Furthermore, the computation burden of resource-poor devices is low in our scheme by outsourced decryption. Besides, we presented the security proof for our scheme and evaluated its precise performance by conducting extensive experimental simulations to demonstrate its efficiency and practicality in data sharing of smart city applications. In addition, although our proposal is designed for data sharing of applications in smart city, it can also be utilized for more generic systems.

## Data Availability

The data sets used and/or analyzed during the current study are available from the corresponding author upon reasonable request.

## References

[1] Zhang C (2020). Design and application of fog computing and internet of things service platform for smart city. Future Gener. Comput. Syst..

[2] Miao Y, Liu X, Choo K-KR, Deng RH, Wu H, Li H (2019). Fair and dynamic data sharing framework in cloud-assisted internet of everything. IEEE Internet Things J..

[3] Y. Wu, X. Wang, W. Susilo, G. Yang, Z.L. Jiang, Q. Chen, P. Xu, Efficient server-aided secure two-party computation in heterogeneous mobile cloud computing. *IEEE Trans. Depend. Secure Comput.* (2020)

[4] Y. Zhang, P. Wang, H. Huang, Y. Zhu, D. Xiao, Y. Xiang, Privacy-assured fogcs: chaotic compressive sensing for secure industrial big image data processing in fog computing. *IEEE Trans. Ind. Inform.* (2020)

[5] Riasudheen H, Selvamani K, Mukherjee S, Divyasree I (2020). An efficient energy-aware routing scheme for cloud-assisted manets in 5g. Ad Hoc Netw..

[6] J. Wei, X. Chen, X. Huang, X. Hu, W. Susilo, Rs-habe: revocable-storage and hierarchical attribute-based access scheme for secure sharing of e-health records in public cloud. IEEE Trans. Depend. Secure Comput. PP(99), 1 (2019)

[7] V. Goyal, O. Pandey, A. Sahai, B. Waters, Attribute-based encryption for fine-grained access control of encrypted data, in *Proceedings of the 13th ACM Conference on Computer and Communications Security* (ACM, 2006), pp. 89–98

[8] G. Wang, Q. Liu, J. Wu, Hierarchical attribute-based encryption for fine-grained access control in cloud storage services, in *Proceedings of the 17th ACM Conference on Computer and Communications Security*, pp. 735–737 (2010)

[9] J. Li, Y. Zhang, J. Ning, X. Huang, G.S. Poh, D. Wang, Attribute based encryption with privacy protection and accountability for cloudiot. *IEEE Trans. Cloud Comput.* (2020)

[10] Rasori M, Perazzo P, Dini G (2020). A lightweight and scalable attribute-based encryption system for smart cities. Comput. Commun..

[11] P. Zhang, Z. Chen, K. Liang, S. Wang, T. Wang, A cloud-based access control scheme with user revocation and attribute update, in *Australasian Conference on Information Security and Privacy* (Springer, 2016), pp. 525–540

[12] J. Hong, K. Xue, Y. Xue, W. Chen, D.S. Wei, N. Yu, P. Hong, Tafc: Time and attribute factors combined access control for time-sensitive data in public cloud. *IEEE Trans. Serv. Comput.* (2017)

[13] Fan K, Wang J, Wang X, Li H, Yang Y (2018). Secure, efficient and revocable data sharing scheme for vehicular fogs. Peer-to-Peer Netw. Appl..

[14] Premkamal PK, Pasupuleti SK, Alphonse P (2019). Efficient revocable cp-abe for big data access control in cloud computing. Int. J. Secur. Netw..

[15] A. Sahai, B. Waters, Fuzzy identity-based encryption, in *Annual International Conference on the Theory and Applications of Cryptographic Techniques* (Springer, 2005), pp. 457–473. Springer

[16] Jiang Q, Khan MK, Lu X, Ma J, He D (2016). A privacy preserving three-factor authentication protocol for e-health clouds. J. Supercomput..

[17] Q. Jiang, N. Zhang, J. Ni, J. Ma, K.K.R. Choo, Unified biometric privacy preserving three-factor authentication and key agreement for cloud-assisted autonomous vehicles. *IEEE Trans. Veh. Technol.* PP(99), 1 (2020)

[18] Z. Zhang, P. Zeng, P. Pan, K.-K.R. Choo, Large-universe attribute-based encryption with public traceability for cloud storage. *IEEE Internet Things J.* (2020)

[19] Sethi K, Pradhan A, Bera P (2020). Practical traceable multi-authority cp-abe with outsourcing decryption and access policy updation. J. Inf. Secur. Appl..

[20] P.K. Premkamal, S.K. Pasupuleti, P. Alphonse, Dynamic traceable cp-abe with revocation for outsourced big data in cloud storage. *Int. J. Commun. Syst.*, 4351 (2020)

[21] J. Zhang, T. Li, M.S. Obaidat, C. Lin, J. Ma, Enabling efficient data sharing with auditable user revocation for IOV systems. *IEEE Syst. J.* (2021)

[22] J. Li, Y. Wang, Y. Zhang, J. Han, Full verifiability for outsourced decryption in attribute based encryption. IEEE Trans. Serv. Comput. (2017)

[23] H. Wang, D. He, J. Han, Vod-adac: anonymous distributed fine-grained access control protocol with verifiable outsourced decryption in public cloud. *IEEE Trans. Serv. Comput.* (2017)

[24] H. Nasiraee, M. Ashouri-Talouki, Anonymous decentralized attribute-based access control for cloud-assisted IoT. *Future Gener. Comput. Syst.* (2020)

[25] Premkamal PK, Pasupuleti SK, Alphonse P (2019). A new verifiable outsourced ciphertext-policy attribute based encryption for big data privacy and access control in cloud. J. Ambient Intell. Human. Comput..

[26] Fu X, Nie X, Wu T, Li F (2018). Large universe attribute based access control with efficient decryption in cloud storage system. J. Syst. Softw..

[27] Zhang P, Chen Z, Liu JK, Liang K, Liu H (2018). An efficient access control scheme with outsourcing capability and attribute update for fog computing. Future Gener. Comput. Syst..

[28] R.L. Rivest, A. Shamir, D.A. Wagner, Time-lock puzzles and timed-release crypto (1996)

[29] E. Androulaki, C. Soriente, L. Malisa, S. Capkun, Enforcing location and time-based access control on cloud-stored data, in *2014 IEEE 34th International Conference on Distributed Computing Systems* (IEEE, 2014), pp. 637–648

[30] L. Li, Z. Wang, N. Li, Efficient attribute-based encryption outsourcing scheme with user and attribute revocation for fog-enabled IoT. *IEEE Access* (2020)

[31] Wang H, Zheng Z, Wu L, Li P (2017). New directly revocable attribute-based encryption scheme and its application in cloud storage environment. Cluster Comput..

[32] J.K. Liu, T.H. Yuen, P. Zhang, K. Liang, Time-based direct revocable ciphertext-policy attribute-based encryption with short revocation list, in *International Conference on Applied Cryptography and Network Security* (Springer, 2018), pp. 516–534

[33] Xiong H, Zhao Y, Peng L, Zhang H, Yeh K-H (2019). Partially policy-hidden attribute-based broadcast encryption with secure delegation in edge computing. Future Gener. Comput. Syst..

[34] H. Aqeel, S.T. Ali, A provable and user revocable ciphertext-policy attribute-based encryption with updatable ciphertext, in *Innovations in Computer Science and Engineering* (Springer, 2019), pp. 391–399

[35] Lee K (2016). Self-updatable encryption with short public parameters and its extensions. Designs Codes Cryptogr..

[36] H. Cui, R.H. Deng, Y. Li, B. Qin, Server-aided revocable attribute-based encryption, in *European Symposium on Research in Computer Security* (Springer, 2016), pp. 570–587

[37] Xu S, Yang G, Mu Y (2019). Revocable attribute-based encryption with decryption key exposure resistance and ciphertext delegation. Inf. Sci..

[38] Qin B, Zhao Q, Zheng D, Cui H (2019). (dual) server-aided revocable attribute-based encryption with decryption key exposure resistance. Inf. Sci..

[39] Wei J, Liu W, Hu X (2018). Secure and efficient attribute-based access control for multiauthority cloud storage. IEEE Syst. J..

[40] Li J, Ren K, Kim K (2009). A2be: Accountable attribute-based encryption for abuse free access control. IACR Cryptol. ePrint Arch..

[41] J. Li, Q. Huang, X. Chen, S.S. Chow, D.S. Wong, D. Xie, Multi-authority ciphertext-policy attribute-based encryption with accountability, in *Proceedings of the 6th ACM Symposium on Information, Computer and Communications Security*, pp. 386–390 (2011)

[42] W. Zhang, Y. Wu, Z. Zhang, H. Xiong, Z. Qin, Multi-authority Ciphertext-policy Attribute Based Encryption with Accountability (2020). arXiv preprint arXiv:2009.04748

[43] Z. Liu, Z. Cao, D.S. Wong, Blackbox traceable cp-abe: how to catch people leaking their keys by selling decryption devices on ebay, in *Proceedings of the 2013 ACM SIGSAC Conference on Computer and Communications Security*, pp. 475–486 (2013)

[44] A. Kiayias, Q. Tang, How to keep a secret: leakage deterring public-key cryptosystems, in *Proceedings of the 2013 ACM SIGSAC Conference on Computer and Communications Security*, pp. 943–954 (2013)

[45] Ning J, Dong X, Cao Z, Wei L, Lin X (2015). White-box traceable ciphertext-policy attribute-based encryption supporting flexible attributes. IEEE Trans. Inf. Forens. Secur..

[46] Liu Z, Duan S, Zhou P, Wang B (2019). Traceable-then-revocable ciphertext-policy attribute-based encryption scheme. Future Gener. Comput. Syst..

[47] Yan X, He X, Yu J, Tang Y (2019). White-box traceable ciphertext-policy attribute-based encryption in multi-domain environment. IEEE Access.

[48] Zhang K, Li H, Ma J, Liu X (2018). Efficient large-universe multi-authority ciphertext-policy attribute-based encryption with white-box traceability. Sci. China Inf. Sci..

[49] A. De Caro, V. Iovino, jpbc: Java pairing based cryptography, in *Proceedings of the 16th IEEE Symposium on Computers and Communications, ISCC 2011, Kerkyra, Corfu, Greece, June 28–July 1*, pp. 850–855 (2011)

